# Toward MR protocol-agnostic, unbiased brain age predicted from clinical-grade MRIs

**DOI:** 10.1038/s41598-023-47021-y

**Published:** 2023-11-10

**Authors:** Pedro A. Valdes-Hernandez, Chavier Laffitte Nodarse, Julio A. Peraza, James H. Cole, Yenisel Cruz-Almeida

**Affiliations:** 1 Department of Community Dentistry and Behavioral Science, University of Florida, 1329 SW 16th Street, Ste. 5180, Gainesville, FL 32610 USA; 2 Pain Research and Intervention Center of Excellence, University of Florida, Gainesville, FL USA; 3 Center for Cognitive Aging and Memory, McKnight Brain Institute, University of Florida, Gainesville, FL USA; 4 Department of Physics, Florida International University, Miami, FL USA; 5 Department of Computer Science, Centre for Medical Image Computing, University College London, London, UK; 6 Dementia Research Centre, Queen Square Institute of Neurology, University College London, London, UK; 7 Department of Neuroscience, College of Medicine, University of Florida, Gainesville, USA

**Keywords:** Data mining, Image processing, Machine learning, Statistical methods, Predictive medicine, Brain imaging, Magnetic resonance imaging

## Abstract

The difference between the estimated brain age and the chronological age (‘brain-PAD’) could become a clinical biomarker. However, most brain age models were developed for research-grade high-resolution T1-weighted MRIs, limiting their applicability to clinical-grade MRIs from various protocols. We adopted a dual-transfer learning strategy to develop a model agnostic to modality, resolution, or slice orientation. We retrained a convolutional neural network (CNN) using 6281 clinical MRIs from 1559 patients, among 7 modalities and 8 scanner models. The CNN was trained to estimate brain age from synthetic research-grade magnetization-prepared rapid gradient-echo MRIs (MPRAGEs) generated by a ‘super-resolution’ method. The model failed with T2-weighted Gradient-Echo MRIs. The mean absolute error (MAE) was 5.86–8.59 years across the other modalities, still higher than for research-grade MRIs, but comparable between actual and synthetic MPRAGEs for some modalities. We modeled the “regression bias” in brain age, for its correction is crucial for providing unbiased summary statistics of brain age or for personalized brain age-based biomarkers. The bias model was generalizable as its correction eliminated any correlation between brain-PAD and chronological age in new samples. Brain-PAD was reliable across modalities. We demonstrate the feasibility of brain age predictions from arbitrary clinical-grade MRIs, thereby contributing to personalized medicine.

## Introduction

Brain age predicted from brain Magnetic Resonance Images (MRIs) using machine learning methods^1–3^ has the potential to be a biomarker of disease^4–14^. The difference between the predicted brain age and the chronological age, namely, the ‘brain-PAD’ or ‘brain age gap’, has been shown to be sensitive to pathologies or good lifestyle factors. People with higher brain-PAD values are more likely to have a disease or be at risk of developing a disease; whereas people with lower brain-PAD values are more likely to be healthier^2,4,7,11,15–21^.

Most brain age prediction methods were trained with, and are thus better suitable for, high-resolution (near isotropic 1 mm voxel size) three-dimensional (3D) brain T1-weighted (T1w) MRIs primarily obtained for research purposes, such as the magnetization-prepared rapid gradient-echo (MPRAGE) or the spoiled gradient recalled (3D-SPGR) sequences. This limits the clinical applicability of these methods since the models trained on high-resolution high-quality research scans may not generalize well to the typical brain MRIs data acquired at scale in a myriad of hospitals across the world. For example, a clinical brain MR scanning session could only include a fast two-dimensional (2D) T1w, or non-T1 modalities like the T2-weighted (T2w) or the Fluid Attenuation Inversion recovery (FLAIR), with good axial, coronal or sagittal in-plane resolution but poor slice resolution (e.g., thickness ~ 5–10 mm). Therefore, brain age prediction models that can deal with MRIs acquired using any clinical MR protocol (i.e., clinical-grade MRIs) are needed. A good start in the development of such models for its application in case–control studies requiring group-level analyses is that they at least perform similarly to those using research-grade T1w MRIs; whereas higher accuracy will be eventually needed for personalized medicine applications, e.g., using brain-PAD as a biomarker of disease.

There have been some efforts to deal with non-T1w modalities in the literature. Cole^22^ used six modalities as features to train a brain age model in 2205 participants of the UK Biobank. With the same goal, Rokicki et al.^20^ used several T1w-based morphometric features and the T1w/T2w ratio in 750 healthy participants and Millar et al.^8^ used the structural and functional MRIs of 390 healthy participants. However, the former study advocates for the use of all modalities combined as features for brain age prediction, while the latter studies were trained with relatively small sample sizes. Moreover, both studies used research-grade MRI databases. An exception of the use of research-grade MRIs is Wood et al.^23^ who used clinical-grade T2 weighted (T2w) and diffusion-weighted images (DWI) were used to train a convolutional neural network (CNN) to predict brain age. However, the technique is restricted to axial MRIs of these two modalities. In general, a main problem of achieving a model that can deal any MRI, whether research-graded or clinical-graded, is that it needs to be trained using a great amount of MRIs covering the widest possible spectrum of MR protocols.

Here, we explore an alternative approach leveraging existing T1w-based brain aging methods to develop a brain age prediction method that could deal with any modality, resolution, or slice orientation. To achieve that, we advocate for the transfer of what super-resolution (SR) methods learned about the relationship between clinical-grade and research-grade MRIs. In particular, we incorporate a preprocessing step before brain age prediction that is the prediction of a research-grade 1-mm isotropic MPRAGE from the clinical MRI using the “Synthetic SR” (SynthSR)^24^. SynthSR, publicly available in FreeSurfer, was trained using the concatenation of a U-Net regression and a U-net segmentation on a dataset of 1-mm isotropic 3D MPRAGE scans and companion neuroanatomical labels. It predicts a synthetic MPRAGE from an input neuroimage of any modality, orientation, or resolution with enough quality to produce morphometric results similar to those obtained when using the actual MPRAGE.

To predict brain age from either actual or synthetic MPRAGEs, we used DeepBrainNet^2^. This is a publicly available CNN trained on a research-grade T1w dataset of 11,729 participants from 18 studies spanning different scanners, ages, and locations. We chose DeepBrainNet because its predictions appeared to be sensible to clinical conditions^2,6^, making it a promising biomarker of disease. Here, we specifically repurposed DeepBrainNet via transfer learning retraining on the actual and synthetic MPRAGEs predicted from 6224 clinical MRIs, distributed among 7 different modalities and 8 different models of scanners, from 1540 patients that were scanned at more than 15 facilities of the University of Florida (UF) Health System. This transfer learning approach reduces the large image dataset requirement for training the model from scratch.

With this double transfer learning approach, we met midway between leveraging what SynthSR and DeepBrainNet have learned, making the latter potentially agnostic to the modality, resolution, or clinical nature of the MRI. Our first hypothesis is rather comparative: that the predictive accuracy of our retrained model applied to clinical-grade MRIs from any MR protocol would be comparable to the accuracy that has been reported when using DeepBrainNet on research-grade MPRAGEs^2^. We also assessed how the transfer learning retraining of DeepBrainNet improved prediction using our clinical data. To that end, we tested the null hypothesis that the accuracies of the original DeepBrainNet model and our retrained version were equal when applied to the same testing data. Finally, we also examined whether the retrained DeepBrainNet model performed equally on actual and synthetic MPRAGEs, also by testing the null hypothesis that their accuracies were equal in our data.

When predicting brain age, a bias consisting of an overestimation of younger ages and an underestimation of older ages greatly reduces accuracy. This is the so-called “regression toward the mean” phenomenon^25,26^, and/or “regression dilution”^27,28^ due to errors in the features (i.e., the MRIs) used to predict the outcome (i.e., brain age). As long as the bias is linear and not severe in test samples, it can be accounted for by regressing out chronological age from brain-PAD in group-level analysis^29^. But there are situations in which we may also desire to explicitly obtain bias-corrected brain ages or brain-PADs. For example, (a) when unbiased summary statistics (e.g., the mean) of brain-PAD are evaluated to understand group differences in brain-PAD in terms of accelerated brain age or brain neoteny within the groups; or (b) when the brain-PAD of a test individual is used as a personalized biomarker of disease (provided that the model is accurate enough to serve for this clinical purpose). However, a correction formula depends on some functional form of the bias in the sample, and, for samples that are too small in situation (a), or in situation (b) altogether, we cannot estimate the bias reliably because a minimum sample size is required for a robust linear regression fit. Thus, a model of the bias would have to be determined in a sufficiently large sample, independent from both the sample used to train the brain age prediction model and the test sample, namely the “bias estimation set”, so the parameters of the bias model could be used to correct the brain ages in the test samples, provided that the bias is generalizable.

Here, we set out to characterize the bias in brain age of our clinical-grade MRI data. To that end, we proposed, fitted, and selected the best among several putative linear models of the bias, including those with a moderation by modality and/or scanner model of the age-related slope. At the expense of a potential loss in prediction accuracy, we then proposed a correction method based on the parameters of the selected bias model that do not inflate prediction accuracy, contrary to some correction efforts in the literature^28^. Bias model selection was based on the minimization of the mean absolute error (MAE) of the bias-corrected brain age in evaluation sets during cross-validation. We hypothesized that the bias is generalizable, thus allowing its removal from a test sample independent from the bias training set used to quantify the bias, “removal” meaning no significant correlation between the bias-corrected brain-PAD and chronological age. We also hypothesized that the bias-corrected brain-PAD would be consistent across modalities and repeat scans within subjects.

The current paper is a proof-of-concept on the pursuit of strategies that synthesize MRIs obtained under arbitrary clinical setups to ready them for brain age predictions. Our current aim is not to achieve the accuracy needed for personalized medicine (a long-term aspiration, though), and no brain age method has that capability so far. Instead, we aim to show that, under the above-proposed harmonization of clinical MRIs, a prediction performance similar to that shown by brain age models in research-grade MRI data may be reached, and that these predictions are reliable across varied clinical MR protocols. This is a modest, but innovative early step toward the development of brain age-based biomarkers based on MRI data available in healthcare systems.

## Results

### Sample distribution across modalities and scanner models

This study used MRIs from a randomly selected initial sample of subjects that were scanned between February 2017 and March 2021 at more than 15 facilities of the UF Health System. We received the raw DICOMS from 24,732 MRIs of 1727 patients. After removing all non-brain and partial-brain MRIs, we ended up with a total of 7062 brain MRIs distributed among 8 modalities and repetitions. After quality control (QC) and synthetic MPRAGE prediction, we eliminated 781 MRIs, including three T1w Gradient Echo MRIs for being a modality with too few samples, to obtain a final sample of 6281 whole-brain MRIs across 7 modalities from 1559 patients. Table 1 shows how these MRIs are distributed among the 7 modalities, 8 scanner models and repetitions. The modalities were the actual 3D MPRAGE, and the 2D MRIs: T1w and T2-weighted (T2w), T1w and T2w Fluid attenuated inversion recovery (FLAIR), T1w and T2w Gradient Echo (GRE) and Inversion Recovery (IR). Table S1 in the Supplemental Materials evidences the high variability in some of the MRI parameters and slice orientations in the sample. The sample had 1039 females and 501 males with chronological age ranging from 15 to 95 years, with a mean, median, and standard deviation of 53.5, 56 and 18 years.

**Table 1:** Distribution of MRIs that passed QC among modalities and scanners.

Modality	Scanner								Total	Number of repetitions			
	Aera	Avanto	Prisma	Sola	Signa HDxt	Skyra	Titan 3T	Verio		1	2	3	4
MPRAGE	157	208	169	49	89	31	38	199	940	916	12	0	0
2D T1w	261	504	234	32	157	31	115	435	1769	675	526	10	3
2D T2w	399	565	297	48	270	44	124	474	2221	601	756	32	3
2D T1wFLAIR	2	8	2	0	73	1	0	4	90	50	20	0	0
2D T2wFLAIR	231	118	226	48	151	38	87	110	1009	971	19	0	0
2D T2wGRE	4	13	36	1	0	17	0	27	98	34	32	0	0
2D IR	0	0	0	0	154	0	0	0	154	154	0	0	0
Total	1054	1416	964	178	894	162	364	1249	6281	3469	1365	42	6

The first section shows the number of MRIs per modality and scanner. The second section shows the number of subjects having 1, 2, 3 or 4 repetitions of each modality.

### Predicted MPRAGEs

**Figure 1:**
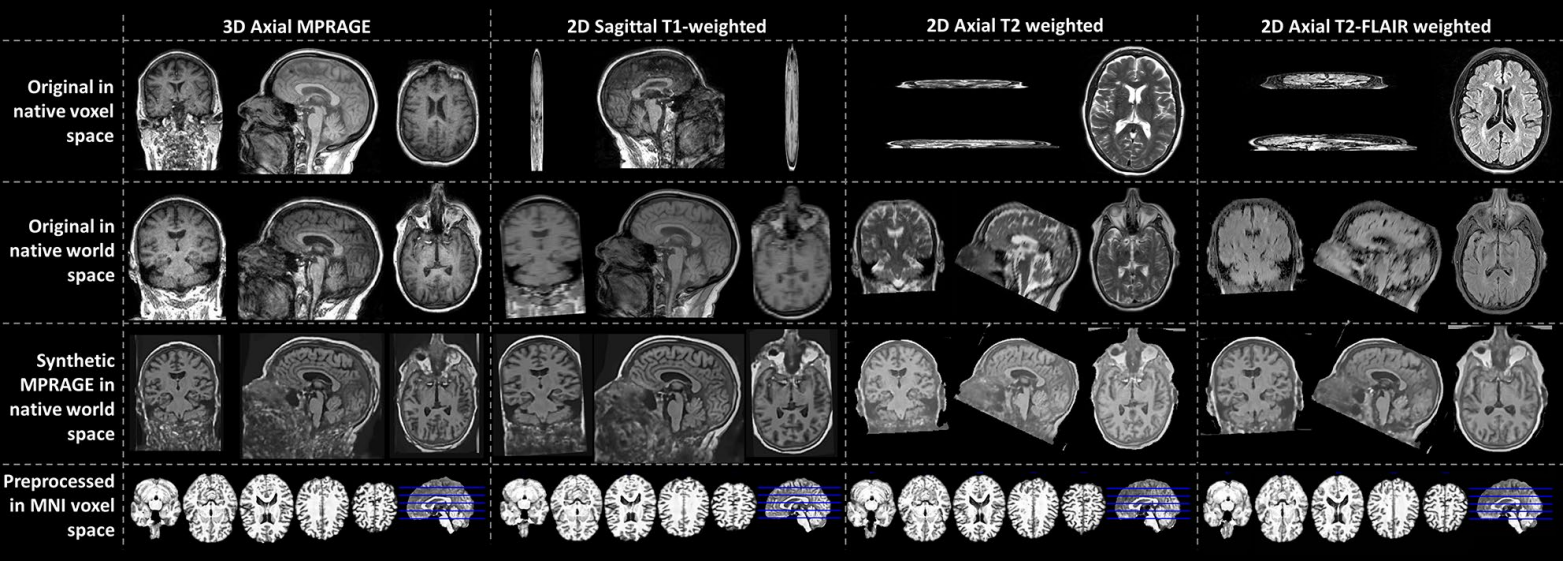
Predicted MPRAGEs for three of the five modalities included in this study. The data belongs to a single participant. The top row shows selected slices in the slice, phase-encoding and readout directions (left, right top and right bottom, respectively). The bottom row shows the bottommost (z =  − 27 mm, slice 46), topmost (z = 53 mm, slice 126), and three intermediate slices (z =  − 7, 13, and 33 mm, slices 66, 86 and 106, respectively) of the 80 slices used for brain age prediction. To show images in world space, trilinear interpolation was used. *FLAIR* fluid attenuated inversion recovery.

Figure 1 shows SynthSR in action for some of the MRI modalities of a participant of the sample. In the figure, the first two rows expose the poor resolution of the clinical-grade MRIs along the slice direction. The third row shows how SynthSR remedies this, and the fourth shows the final preprocessed MRI, in the Montreal Neurological Institute (MNI) space, required by DeepBrainNet to predict brain age.

### Brain age predictions

A DeepBrainNet model consists of a two-dimensional (2D) CNN connected to a dense layer with 1024 units with a Rectified Linear Unit (ReLU) activation, an 80% dropout layer (when training), and a single output layer with linear activation (the predicted brain age)^2^. In the original paper^2^, DeepBrainNet models were trained with research-grade high-resolution T1ws to predict brain age independently from 80 selected slices of an MRI in the MNI space. The whole brain age is then calculated as the median across slices.

In this paper, we instead used the actual (i.e., not transformed by SynthSR) clinical-grade MPRAGEs and the synthetic MPRAGEs predicted from all clinical-grade MRIs (i.e., actual MPRAGEs and other modalities) of 71% of the participants of our sample (i.e., the “training set”, 1109 participants, 4770 MRIs) to retrain two CNNs of the DeepBrainNet model: the “InceptionResNetV2” and the “Visual Geometry Group” network with 16 layers (VGG16)^30^. This retraining was also performed under several configurations of hyper-parameters. Furthermore, a bias correction was then added to the top layer of the retrained model so it could output two brain age predictions: the bias-uncorrected and the bias-corrected. We considered several putative models of the bias with the generic form $brain\, age={f}_{{\varvec{\beta}}}\left(chronological \,age\right)+error$, where ${f}_{{\varvec{\beta}}}\left(chronological\, age\right)$ is a function of $chronological \,age$ that could include interactions with modality or scanner model, and that depends on a set of regression coefficients in the vector ${\varvec{\beta}}$_**.**_ We proposed $corrected \,brain\, age={f}_{\widehat{{\varvec{\beta}}}}^{-1}(brain\, age)$, an approach that does not inflate brain age prediction accuracy because ${f}_{\widehat{{\varvec{\beta}}}}^{-1}(brain \,age)$ does not explicitly depend on $chronological\, age$^28^.

To select the optimal combination of CNN (InceptionResNetV2 or VGG16), hyper-parameter configuration (batch size, learning rate, weighting strategies on the observations) and bias model (${f}_{{\varvec{\beta}}}\left(chronological\, age\right)$), we used three-fold cross-validation, stratifying the data at the participant’s level, as detailed below. For each CNN and combination of hyper-parameters, at each iteration of the cross-validation, two folds of the training participants (the “training set of the iteration”) were used to fit the parameters of the CNN. The third fold was split into two subsets. One subset of this third fold (the “bias estimation set of the iteration”) was used to fit, using either weighted least squares (WLS) or ordinary least squares (OLS), several candidate models of the bias. The other subset of the third fold (the “evaluation set of the iteration”) was used to predict the out-of-sample brain ages and evaluate the bias-uncorrected and bias-corrected MAE.

The combination of the CNN and hyper-parameter configuration that minimized the bias-uncorrected MAE, averaged across folds, included the VGG16 model, with a batch size of 80 slices, learning rate of 7e−5 and weighting of the observations proportional to the number of MRIs each participant had across modalities and repetitions. On the other hand, the bias model that minimized the bias-corrected MAE, averaged across folds, was ${f}_{{\varvec{\beta}}}\left(chronological \,age\right)=intercept+slope\times chronological\, age,$ fitted using Ordinary least squares (OLS). Thus, the correction formula was $corrected\, brain\, age=\frac{brain\, age-intercept}{slope}$. In fact, this selected bias model yielded an average bias-corrected MAE that was equal (though less variable), to the decimal place, to the model including a moderation by modality. Thus, we selected the simplest and more stable bias model.

In a first stage of the training for each cross-validation iteration, we loaded the original DeepBrainNet model, set the weights of the fully connected and output layers as trainable, while the weights of the rest of the network were frozen, and trained the model using a maximum of 20 epochs. In most of the iterations the algorithm stopped between the 15th and 20th epoch due to early stopping criteria defined to avoid overfitting. In a second stage of the iteration, we unfroze all the layers of the model and re-trained it with another maximum of 20 epochs. Notably, the algorithm stopped just after the first, second or third epoch due to overfitting, indicating that the retraining was mostly needed in the upper layers.

Finally, the selected CNN model and hyper-parameter configuration were used to train a final model on 93.4% of the participants of the whole training set (with 1036 participants and 4447 MRIs), dedicating 6.6% (the “evaluation set”, with 73 participants and 323 MRIs) to monitor overfitting (i.e., 66.5% and 4.7% of the whole set of participants, respectively). Of the remaining participants who were not in the training set, 12% of the whole sample (the “bias estimation set”, with 186 participants and 841 MRIs) was used to estimate the final parameters of the selected bias model—the intercept and slope were 15.2 years and 0.7, respectively.

Stratification was done at the participant’s level to avoid spurious over-performance due to “participant leakage”. We also tried to achieve similar distributions of chronological age, sex, modalities and scanner models among sets. The training and bias estimation sets only included MRIs of four (i.e., MPRAGEs, T1ws, T2ws, and T2wFLAIRs) out of the seven modalities in the total sample. We excluded the other three modalities (i.e., T1wFLAIRs, T2wGRE and IRs) from the training and bias estimation sets because there were not enough participants with these modalities to guarantee a robust training or linear fit, respectively (see Table 1). Since splitting was done at the participant’s level, the entire data of a participant with at least one MRI of one of the three excluded modalities was not included the training and bias estimation sets. As a consequence, most MRIs acquired using the “Signa HDxt” and “Skyra” scanners were also excluded from the training and bias estimation sets. This forced us to also move all participants that had at least one MRI acquired in any of these two scanner models out of the training and bias estimation sets. This process resulted in a final training and bias estimation sets that represented the above-mentioned 71% and 12% of the whole sample, respectively, with MRIs among four modalities and six scanner models. The remaining 17% (i.e., the “testing set”, with 264 participants and 1514 MRIs) had MRIs among all seven modalities and eight scanner models, and was used to report the generalization error (bias-uncorrected and bias-corrected MAEs). This allowed for the evaluation of the generalization error not only in new participants already represented in the training sample (in-modality and in-scanner) but also those with modalities or scanner models “unseen” by the retrained model (out-modality and out-scanner). Details about the distribution of MRIs in these divisions can be found in Tables S2–S15 of the Supplemental Materials.

**Figure 2:**
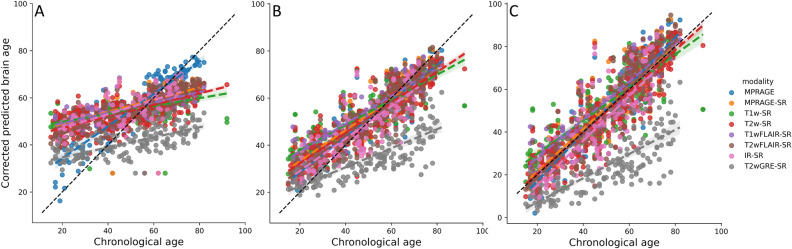
Brain age prediction using the selected CNN (VGG16-based DeepBrainNet) for the MRIs of the testing held-out sample. (**A**) Predictions using the original (un-retrained) model, (**B**) predictions using the retrained model and (**C**) bias-corrected predictions using the retrained model. The colored lines represent the slope of the linear relation between the chronological and the predicted brain age for each modality. *MPRAGE* magnetization-prepared rapid gradient-echo, *T1w* T1-weighted, *T2w* T2-weighted, *FLAIR* fluid attenuated inversion recovery, *GRE* gradient echo, *IR* inversion recovery, *[Modality]-SR* super-resolution synthetic MPRAGE version of [modality].

Figure 2A shows the brain age predictions using the selected, but original (i.e., before being retrained) DeepBrainNet model in the testing set. This figure illustrates the need of retraining when dealing with synthetic MPRAGEs, since this original model performed very poorly on them. Figure 2B shows the bias-uncorrected brain age predictions using the selected retrained DeepBrainNet model in the testing set. The bias in the predictions, that overestimate younger ages and underestimates older ages, is visibly exposed by the slope of the linear relation between the chronological and predicted brain ages.

To test our first group of hypotheses, related to the accuracy of the methods, we summarize the measures of accuracy related to Fig. 2A,B in Table 2, excluding those related to the application of the original DeepBrainNet model on the synthetic MPRAGEs due to its obvious great underperformance (MAE ~ 11–13 years). When using the retrained model on our actual clinical-grade MPRAGEs (see top row of Table 2), the MAE (and bootstrapped CI) was rather high compared to the value reported by Bashyam et al.^2^ when using DeepBrainNet on research-grade high-resolution 3D T1w MRIs, i.e., 5.86 [4.86, 7.06] years versus 4.12 years. Other measures of accuracy of the retrained model on our actual clinical-grade MPRAGEs were the correlation between the predicted brain age and the chronological age (r = 0.92 [0.87, 0.95]) and the coefficient of determination (R^2^ = 0.70 [0.55, 0.81]). These measures of accuracy were not reported by Bashyam et al.^2^ for a test sample.

**Table 2:** Measures of accuracy in the brain age prediction using the selected CNN (VGG16-based DeepBrainNet) for each modality in the testing held-out sample.

CNN model	Modality	Testing sample MAE		Correlation		R^2^		Within-subject difference with original MPRAGE		$\widehat{\text{MAE}}$	
		Mean	95% CI	Mean	95% CI	Mean	95% CI	Mean	95% CI	Mean	95% CI
Retrained VGG16	MPRAGE	5.86	[4.86, 7.06]	0.92	[0.87, 0.95]	0.70	[0.55, 0.81]			5.90	[4.42, 7.41]
	Synthetic MRIs										
	MPRAGE	6.89	[5.70, 8.20]	0.88	[0.81, 0.92]	0.51	[0.29, 0.68]	0.96	[− 0.63, 2.55]	6.86	[5.39, 8.34]
	T1w	8.81	[7.33, 10.72]	0.82	[0.71, 0.89]	0.03	[− 0.44, 0.45]	3.12	[1.44, 4.8]	9.11	[7.69, 10.53]
	T2w	7.21	[6.13, 8.19]	0.88	[0.83, 0.92]	0.49	[0.31, 0.63]	1.29	[− 0.11, 2.68]	7.22	[6.05, 8.39]
	*T1wFLAIR*	8.59	[6.38, 10.73]	0.92	[0.83, 0.94]	0.21	[− 0.24, 0.58]	3.35	[1.3, 5.41]	9.36	[7.45, 11.27]
	T2wFLAIR	6.81	[5.89, 7.89]	0.90	[0.85, 0.93]	0.62	[0.48, 0.73]	0.89	[− 0.63, 2.41]	6.84	[5.53, 8.15]
	*IR*	7.22	[5.75, 9.93]	0.81	[0.68, 0.92]	0.51	[0.26, 0.81]	0.74	[− 1.25, 2.74]	6.47	[4.58, 8.36]
	***T2wGRE***	**16.67**	**[14.33, 19.14]**	**0.75**	**[0.63, 0.83]**	− **3.47**	**[**− **5.83,** − **1.71]**	**10.75**	**[9.04, 12.46]**	**16.79**	**[15.28, 18.31]**
	All synthetic MRIs except T2wGRE	7.50	[7.30, 8.98]	0.87	[0.81, 0.90]	0.46	[0.07, 0.54]	3.05	[1.81, 4.29]	7.39	[6.39, 8.40]
Original VGG16	MPRAGE	6.83	[5.59, 8.28]	0.89	[0.83, 0.93]	0.52	[0.26, 0.71]	0.96	[− 0.62, 2.53]	6.86	[5.38, 8.34]

The first, second and third columns are the bootstrapped MAE, Correlation and coefficient of determination of the *brain age* = *chronological age* linear model, respectively. The fourth column is the estimated within-subject difference in the corrected brain-PAD between each synthetic MPRAGE using the retrained CNN, or the MPRAGE using the original (un-retrained) CNN, and the original MPRAGE using the trained CNN (in simpler words, the comparison of all rows with the first). The fifth column is the “population” estimated MAE.

*MAE* mean absolute error (in years), *CI* confidence interval (adjusted for multiple comparisons using a Bonferroni corrected α), *MPRAGE* magnetization-prepared rapid gradient-echo, *T1w* T1-weighted, *T2w* T2-weighted, *FLAIR* fluid attenuated inversion recovery, *GRE* gradient echo, *IR* inversion recovery.

T2wGRE results are highlighted in bold font and modalities that were not part of the training set are in italics.

Compared to the retrained DeepBrainNet model, the original one was less accurate when applied to the actual MPRAGEs (see bottom row of Table 2), i.e., MAE = 6.83 [5.59, 8.28] years, r = 0.89 [0.83, 0.93] years and, notably, the R^2^ = 0.52 [0.26, 0.71]. However we found no evidence of a “within-subject difference” in the MAE (see “Methods” section for a description of how this was estimated) when compared with the retrained model: 0.96 [− 0.62, 2.53] years.

Finally, when using the retrained model on the synthetic MPRAGEs (see rows 2–9 of Table 2), accuracy was generally worse, though modalities MPRAGE, T2w, T2wFLAIR and IR showed no significant within-subject difference in MAE with the original MPRAGEs. In particular, the model greatly underperformed for T2wGRE, a modality not used for training. Notably, this modality was obtained at a single facility among the more than 15 facilities of the UFHealth System, and with the Signa HDxt GE Medical Systems 1.5T scanner. Because of this remarkable prediction failure, when summarizing measures of accuracy across all modalities, we excluded the T2wGREs to better characterize performance in the rest of the modalities.

Figure 2C shows the bias-corrected brain age predictions, using the selected retrained DeepBrainNet and selected bias model, in the testing set. Table 3 summarizes the measures of accuracy of these predictions. Note that the benefits offered by the correction come at the expense of a worse prediction accuracy. This is expected because the variance of the bias-corrected brain age is 1/slope^2^ times higher than the variance of the bias-uncorrected one. Table 3 also shows that the predictions for the synthetic MPRAGEs of modalities MPRAGE, T1w, T2w, T1wFLAIR and T2FLAIR showed no significant within-subject difference in bias-corrected MAE with the original MPRAGEs.

**Table 3:** Measures of accuracy in the brain age prediction using the selected retrained CNN (VGG16-based DeepBrainNet) with the linear correction layer for the MRIs for each modality in the testing held-out sample.

Modality	Testing sample MAE		Correlation		R^2^		Within-subject difference with original MPRAGE		$\widehat{\text{MAE}}$	
	Mean	95% CI	Mean	95% CI	Mean	95% CI	Mean	95% CI	Mean	95% CI
MPRAGE	6.69	[5.67, 7.99]	0.92	[0.87, 0.95]	0.84	[0.75, 0.89]			6.78	[5.16, 8.40]
Synthetic MRIs										
MPRAGE	7.06	[5.91, 8.44]	0.88	[0.81, 0.92]	0.78	[0.65, 0.85]	0.29	[− 1.44, 2.01]	7.07	[5.45, 8.69]
T1w	8.16	[6.98, 10.29]	0.82	[0.71, 0.89]	0.61	[0.37, 0.78]	1.58	[− 0.24, 3.4]	8.36	[6.8, 9.92]
T2w	7.15	[6.13, 8.17]	0.88	[0.83, 0.92]	0.78	[0.67, 0.83]	0.44	[− 1.08, 1.95]	7.22	[5.94, 8.5]
*T1wFLAIR*	7.06	[5.03, 8.62]	0.92	[0.83, 0.94]	0.76	[0.61, 0.88]	1.11	[− 1.11, 3.34]	7.9	[5.8, 9.99]
T2wFLAIR	7.31	[6.18, 8.34]	0.90	[0.85, 0.93]	0.80	[0.72, 0.86]	0.64	[− 1.01, 2.29]	7.42	[5.98, 8.86]
*IR*	10.39	[8.99, 13.72]	0.81	[0.68, 0.92]	0.62	[0.44, 0.81]	3.03	[0.87, 5.19]	9.81	[7.72, 11.91]
***T2wGRE***	**26.54**	**[23.84, 29.30]**	**0.75**	**[0.63, 0.83]**	− **3.34**	**[**− **5.52,** − **1.68]**	**19.96**	**[18.1, 21.82]**	**26.74**	**[25.08, 28.4]**
All synthetic MRIs except T2wGRE	7.69	[7.01, 9.11]	0.87	[0.81, 0.90]	0.75	[0.59, 0.79]	3.86	[2.51, 5.22]	7.79	[6.69, 0.90]

The first, second and third columns are the bootstrapped MAE, Correlation and coefficient of determination of the *corrected brain age* = *chronological age* linear model, respectively. The fourth column is the estimated within-subject difference in the corrected brain-PAD between each synthetic MPRAGE and the original MPRAGE, both using the retrained CNN (in simpler words, the comparison of all rows with the first). The fifth column is the “population” estimated MAE.

*MAE* mean absolute error (in years), *CI* confidence interval (adjusted for multiple comparisons using a Bonferroni corrected α), *MPRAGE* magnetization-prepared rapid gradient-echo, *T1w* T1-weighted, *T2w* T2-weighted, *FLAIR* fluid attenuated inversion recovery, *GRE* gradient echo, *IR* inversion recovery.

T2wGRE results are highlighted in bold font and modalities that were not part of the training set are in italics.

To quantify whether the bias was removed in the test data (whether the bias is generalizable), we tested if the slope of the linear fit between the bias-corrected brain-PAD and the chronological age was significantly different from zero, for each modality or for the whole sample (i.e., the average slope across modalities). To that end, we fitted the model $brainPAD\sim modality\times chronological \, age$ (in Wilkinson notation) and found evidence to reject the hypothesis that all slopes were equal to zero (F_8,1498_ = 44.1, p ~ 0). Post-hoc analysis provided evidence that this actually owed, unsurprisingly, to the above-mentioned poor prediction performance with the T2wGRE MRIs. That is, each slope, tested separately, was not significantly different from zero (uncorrected p > 0.05), except for T2wGRE (p ~ 0), nor was the slope averaged across all modalities excluding T2wGRE (p > 0.05).

### Internal consistency of brain age predictions across modalities and repetitions

**Figure 3:**
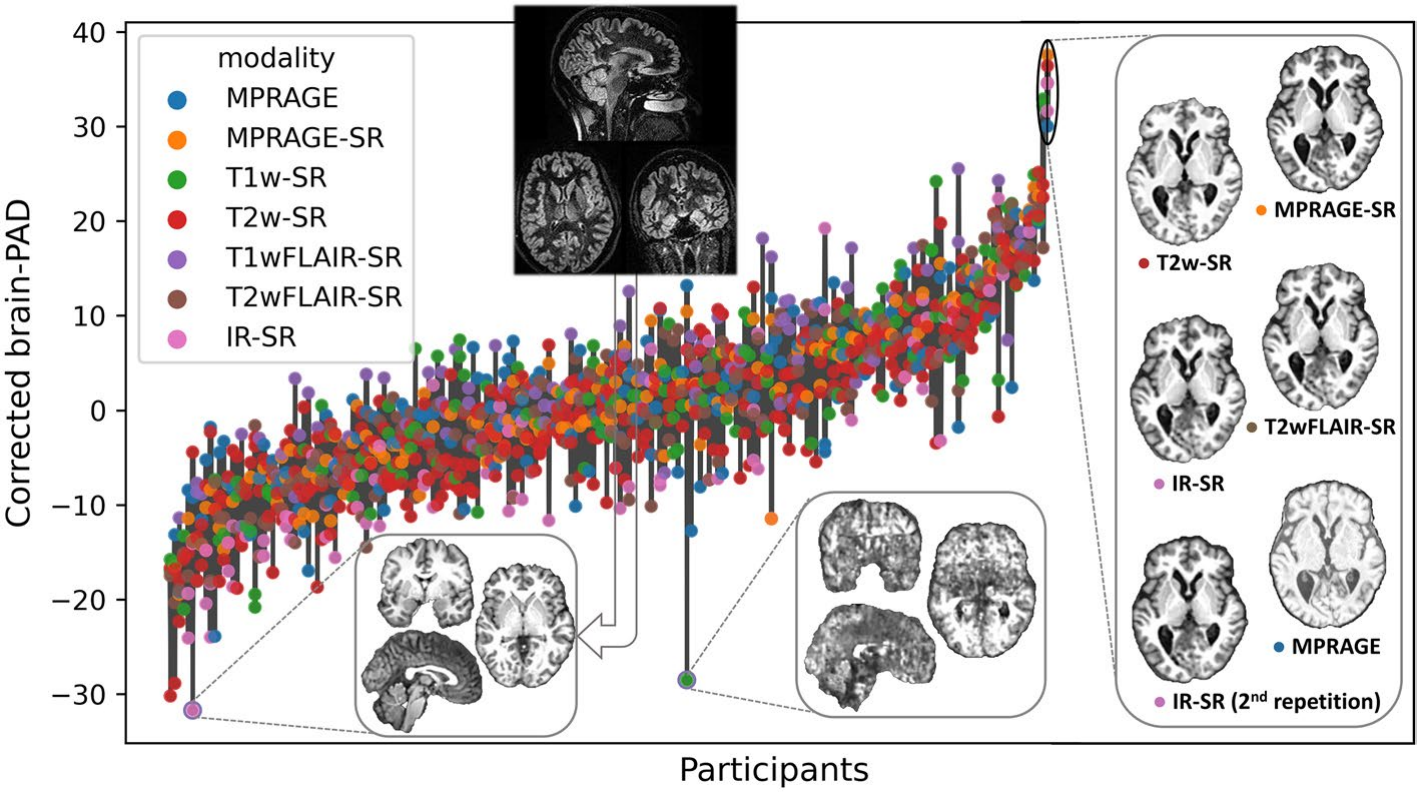
Variability of the bias-corrected predicted brain-PADs for each participant (ordered from the lowest to the highest brain-PAD). The bars represent the range of PAD values within each subject. To illustrate the possible causes of some of the outliers that affect the intra-subject reliability, we show the synthetic MPRAGEs (below the scatter plots) of the two images with bias-corrected brain-PAD farthest from the mean in their own within-subject group. The final image used to estimate brain age, to the left, had no apparent issues. However, it can be appreciated that the corresponding actual MRI (above the scatter plots) is highly noisy. This is because SynthSR is sometimes able to reconstruct any noisy MRI to a synthetic MPRAGE with a relatively good SNR. Thus, QC has to be also done on the actual MRIs. The case to the right had simply such a very poor quality that even SynthSR was not able to recover a meaningful synthetic MPRAGE. This image was not detected by the QC described in this study. Moreover, the inset to the right also shows the actual MPRAGE and synthetic MPRAGEs for the subject having the highest average bias-corrected brain-PAD (26.7 years) in the test set (this participant had no T1wFLAIR or T1w but had two IRs).

To test our hypothesis that the bias-corrected brain-PAD would be consistent across modalities and repeat scans within subjects, we assessed the intra-subject reliability of the predictions. First, we evaluated the Cronbach’s alpha, a measure of internal consistency^31^, on the brain-PAD. Cronbach’s alpha of the bias-corrected brain-PADs was 0.92 with 95% CI of [0.90, 0.93] for the whole testing set and 0.94 with 95% CI of [0.93, 0.95] when excluding the T2wGRE. We also calculated the mean absolute difference between the bias-corrected brain-PADs and their average for each subject. The average of this measure across subjects was 4.4 years with a bootstrapped 95% CI of [4.1, 4.6] years for the whole testing set, 2.5 [2.3, 2.7] years when excluding the T2wGRE MRIs, and 2.0 [1.9, 2.2] years when excluding all modalities that were not used to re-train the model. Figure 3 depicts the brain-PAD for each participant.

## Discussion

In this study, we’ve made significant progress towards establishing the viability of using a clinical-grade MRI, obtained using any MR protocol (e.g., modality, scanner, in-plane resolution or slice thickness or orientation), to predict brain age with convolutional neural networks (specifically DeepBrainNet, retrained with our clinical dataset). This is possible if we utilize the relationship between clinical-grade MRIs and high-resolution research-grade MPRAGEs learned by synthetic reconstruction methods (particularly, SynthSR).

Our first goal was to qualitatively compare the performance of our retrained DeepBrainNet model, when applied to our clinical-grade MRI data, with previous performance reports of the original DeepBrainNet model (for example, by its developers Bashyam et al.^2^). When used with actual MPRAGEs in our test set, the MAE of our retrained model was 5.86 [4.86, 7.06] years. This MAE was higher than the 4.12 years reported by Bashyam et al. when they applied the original model to their test set of research-grade high-resolution T1ws^2^. There could be several reasons for this difference in MAE. Firstly, our higher prediction errors might be linked to various clinical conditions. Our sample size was smaller (166 versus 2739), which could have resulted in an overestimation of the MAE^32^. However, the narrower age range of the participants with MPRAGEs in our test sample (i.e., our 17–82 years versus their 3–93 years) might have offset this^32^.

The MAE is known to be influenced by the distribution of brain age^32^. This is why other accuracy measures, such as the correlation between brain age and chronological age (r), or the coefficient of determination (R^2^; described in the “Methods” section), are often used to supplement accuracy reports and may be more suitable for comparisons across studies^32^. Unfortunately, Bashyam et al.^2^ did not report these two measures for their test data. However, we have reported these measures after using the original DeepBrainNet model on research-grade actual MPRAGEs preprocessed exactly like in the current study^6,33^. In one of these studies, MAE = 6.43 years ([6.07, 6.82]), r = 0.86 ([0.83, 0.87]) and R^2^ = 0.63 ([0.57, 0.68]) for 660 participants with and without chronic musculoskeletal pain^6^. These values indicate that the original DeepBrainNet model, applied to our research pain data, performed (a) better than the same original DeepBrainNet model applied to our clinical-grade actual MPRAGEs (MAE = 6.83 [5.59, 8.28], r = 0.89 [0.83, 0.93], and R^2^ = 0.52 [0.26, 0.71]), but (b) slightly worse than our retrained DeepBrainNet model applied to clinical-grade actual MPRAGEs (MAE = 5.86 [4.86, 7.06], r = 0.92 [0.87, 0.95], and R^2^ = 0.70 [0.55, 0.81]). However, these comparisons should be interpreted with caution because the age range of the participants in that pain study was narrower and more concentrated around the median age than the age range of the current study. Narrower age distributions also tend to favor accuracy when using r or R^2^^32^. Considering the last two paragraphs, future studies with matched samples, in terms of size, age range, demographics and clinical composition, are needed for reliably benchmarking the models.

We also evaluated the performance of our retrained DeepBrainNet model when used with synthetic MPRAGEs (derived from the actual MPRAGE and other modalities). It’s worth noting that the updated model was highly inaccurate for the T2wGREs. The accuracy for the T1ws, T2ws, FLAIRs, and IRs varied between 6.81 and 8.81 years for the MAE, 0.81 and 0.92 for r, and 0.03 and 0.62 for R^2^—the low R^2^ values for the T1ws and T1wFLAIRs were due to their poor alignment with the $brain\, age=chronological \,age$ line. We also compared the performance of our retrained DeepBrainNet model when used with synthetic and actual MPRAGEs. For this, we used a mixed linear model to estimate the within-subject difference in MAEs. We discovered that for some modalities, regardless of whether they were part of the training or not, the difference between the synthetic and actual MPRAGEs was not statistically significant. However, this difference was statistically significant when all synthetic MPRAGEs (excluding the T2wGRE) were combined, with the MAE of the synthetic being 0.96 years higher than that of the actual MPRAGEs.

Our model can handle a wider range of MR protocols than previous models, but this comes at the cost of accuracy. Previous models were limited by the use of only a few modalities and specific acquisition parameters. For instance, Wood et al.^23^ achieved a MAE of less than 4 years by using a model that only works with axial clinical-grade T2w and DWI MRIs together. The accuracy gap between our model and theirs could be also due to the use of different deep learning models. They employed DenseNEt121^34^, a 3D model that had a lower prediction bias than the original DeepBrainNet model. In fact, in a recent revision of the performance of deep learning methods for brain age prediction, available as a preprint^35^, the original DeepBrainNet model showed higher prediction bias than other CNNs. Furthermore, DeepBrainNet depends on preprocessing steps that could cause prediction errors. An incorrect brain extraction in the native space could lead to both wrong brain tissue classification and abnormal shearing when normalizing to the MNI template. Another important factor is that we retrained the model on a sample without detailed clinical information. Although we excluded subjects with noticeable structural abnormalities, some “very unhealthy” participants might still be in our dataset, affecting the model’s definition of a “healthy brain”, which is already compromised by the clinical nature of our data, for a given age or resulting in higher errors when testing. Additionally, as shown in Fig. 3, we could not remove all low-quality MRIs, which affected both training and testing. The last two paragraphs suggest that, while our methodology offers a novel and promising approach to handle various MR modalities, it also has some limitations and room for enhancement (e.g., CNN model, preprocessing, and diversity of MR protocols in the training sample).

Our second set of results pertains to the elimination of a bias that tends to overestimate younger predicted ages and underestimate older ones^25–29^. A bias model, which was independent of modality or scanner model, was chosen using cross-validation. This model was generalizable, as the correction based on the model’s estimated parameters in the bias estimation set resulted in bias-free brain ages in the independent testing set (except for T2wGRE). It’s important to note that for the selected bias model, the variance of bias-corrected brain age of the entire dataset is 1/slope^2^ times the variance of the bias-uncorrected brain age. Therefore, the fact that the bias-corrected MAE was only 6.83–5.89 = 0.94 years higher than the bias-uncorrected MAE for the actual MPRAGEs, and only 7.69–7.50 = 0.19 years higher for all synthetic MRIs excluding T2wGRE, can be seen as a positive indicator of the performance of the bias-corrected brain age predictions. Lastly, as anticipated, the predictions were consistent across modalities and/or repetitions at the participant level (with redundant levels, according to a Cronbach’s alpha of 0.94 [0.93, 0.95], when excluding the T2wGREs), and the average within-subject variability in brain-PAD (the average within-subject MAE) was 2.4 years when excluding the T2wGRs.

One major drawback of this study is that the deployed model is not yet agnostic to modality. Even after correcting the bias, some modalities exhibited a MAE higher than that of the actual MPRAGEs (ranging from 0.89 to 3.35 years, excluding the T2wGREs). The model performed very poorly for the T2wGREs, which were only obtained with the Signa HDxt GE 1.5T at a single site. This could be attributed to various domain mismatches or potential sources of bias. For instance, the T2wGRE modality and the Signa HDxt scanner type were absent from the training data. Moreover, most of our MRI data came from Siemens scanners, which could skew the accuracy in favor of this vendor. Additionally, the procedures or data management at this site could differ significantly from the others. However, these explanations are not consistent with the much better accuracy for other modalities from the same site. We need larger and more diverse training datasets with different clinical MR protocols and scanner vendors to further investigate this issue.

Another limitation is that the MRI data we used in our clinical study may not accurately reflect the variety of scanner models found in hospitals or clinics throughout the United States or internationally. Verifying this could be challenging, as information about the vendor, model, or field strength is not easily accessible. It might require complex meta-analyses of published clinical MRI data, which are probably less plentiful than research MRI data. Our sample also does not include T1w MRIs from other high-resolution sequences frequently used in clinical and research studies, such as the 3D-SPGR sequences, including the fast spoiled gradient echo (FSPGR). However, we anticipate that the retrained DeepBrainNet would perform similarly on both synthetic and actual 3D-SPGRs, given that the original DeepBrainNet was already adapted to this T1w sequence.

The data was divided based on chronological age, sex, modality, and scanner model to ensure that the choice of CNN model and training hyperparameters was not influenced by attempts to “correct” domain mismatches in these variables between the training and evaluation sets during cross-validation. However, this approach had a drawback: the weighting strategy for the participants was solely based on the number of MRIs they had during cross-validation, as the aforementioned variables were already balanced. Consequently, since the distribution of these variables differs between the final training and test sets, the reported generalization error might be higher than if these sets had similar distributions of these variables. This could have been circumvented by disregarding the cross-validation’s recommendation and also weighting based on age, modality, and scanner during the final training. Yet, comparing the two generalization errors in the test data is meaningless in machine learning. Indeed, the comparisons made in the test data in Tables 2 and 3 of different strategies (e.g., using the original model versus retrained, or using actual versus synthetic MPRAGEs) or modalities are merely “suggestive” and not generalizable. However, the statistical tests we conducted on the test data are still insightful and useful for generating new plausible hypotheses, but they still need to be validated with new independent data. In future studies, we will need to choose among different strategies for weighting, domain adaptation, robust modeling, or data augmentation using another, independent training data and evaluate the generalization error with yet another test data.

Moreover, given that we did not have enough information about the clinical characteristics of the patients, we were not able to determine how much of their possible underlying conditions are affecting the estimations. In fact, we did not test whether brain-PAD obtained from a clinical brain MRI is sensitive enough to underlying conditions (dementia, chronic pain, etc.). This should be the next study, provided we can gather enough clinical data from the patients.

In addition, the performance of the model was likely affected by the fact we could not visually inspect all MRIs in the database. First, some MRIs with very poor quality (e.g., noise or artifacts) could have affected training and/or accuracy on the testing set. Likewise, the preprocessing steps could have had the same type of impact in both training and validation. Future study should avoid improper brain extraction to affect the affine normalization by introducing aberrant shearing. A viable route could be to first normalize the whole-head MRI and initialize the brain-extraction algorithm using the FSL’s template brain mask or adopt novel deep learning-based techniques for brain extraction like HD-BET^36^.

In conclusion, it appears feasible to predict brain age from any clinical MRIs from patients that visit the UF’s Health System in FL, USA. Further research is needed to test the generalizability of these predictions to any clinical facility and to investigate the ability of the predicted brain age difference in multimodal clinical data to characterize pathological conditions. We emphasize that clinical settings are where brain age biomarkers (and any biomarker in general) are most needed. We have demonstrated the feasibility of brain age predictions on clinical populations, taking additional steps toward the development of biomarkers in personalized medicine. However, like most of published brain age studies to date, the reported MAE indicates that the model  is still far from being ready to be deployed for this purpose. That is, while it can serve to report of unbiased summary statistics (e.g., the mean) of brain ages in small subsamples of interest, it does not yield individualized predictions with clinical relevance. This limitation is currently common to all brain age methods, irrespective of the MRI protocol used. We recommend novel and more accurate deep learning methods to increase the accuracy of the brain age predictions. Nevertheless, our approach is the first proof-of-concept for the use of super-resolution methods to develop modality- and scanner-agnostic brain age prediction methods. This is a promising route for the development of useful brain age-based clinical tools that can be applied to clinical MRIs, which are known to manifest in a wide variety of contrasts due to the use of arbitrarily customized MR protocols (e.g., with varied Repetition Times, Echo Times, Inversion Times, slice orientation or thickness, averages) for specific clinical purposes.

## Methods

All participants, or their legal guardians, gave informed consent for their clinical data to be used for research purposes. All methods were performed in accordance with the relevant guidelines and regulations. This research was performed in accordance with the declaration of Helsinki. MRI acquisition was carried out after all participants completed a screening form to determine MRI eligibility. We received de-identified MRI data. This study was approved by the Institutional Review Board of the University of Florida (IRB No. 202101469).

### DeepBrainNet architectures for brain age prediction

A DeepBrainNet consists of a CNN connected to a dense layer with 1024 units with a Rectified Linear Unit (ReLU) activation, an 80% dropout layer (when training), and a single output layer with linear activation (the predicted brain age)^2^. The CNN can be the “InceptionResNetV2”^37^ or the “Visual Geometry Group” network with 16 layers (VGG16)^30^, initialized with ImageNet weights before trained with MRI data^2^. Since these CNNs predict from 2D images, DeepBrainNet operates with the 2D slices of an MRI as independent samples. The individual’s predicted brain age is the median of the predictions across a set of slices. DeepBrainNet architectures were trained with preprocessed research-grade T1w images from 11,729 individuals (ages 3–95 years) from various geographic locations, scanners, acquisition protocols, and studies, and tested in an independent sample of 2739 individuals. The required preprocessing for a T1w brain MRI is described in the next section.

### Preprocessing of the MRIs

We preprocessed all actual and synthetic MPRAGEs as required for the use of DeepBrainNet. We skull-stripped them using *smriprep* (https://www.nipreps.org/smriprep/usage.html), i.e., the image was corrected for intensity non-uniformity using *N4BiasFieldCorrection*^38^ distributed with ANTs 2.2.0 and skull-stripped with a Nipype implementation of the *antsBrainExtraction.sh* workflow from ANTs^39^, using OASIS30ANTs as target template. Finally, we transformed the skull-stripped images to the 1-mm isotropic voxel FSL skull-stripped T1w template [“MNI152, LPS orientation (Right → Left, Anterior → Posterior, Inferior → Superior), and 218 × 182 × 218 dimensions] using a 12-parameter linear affine transformation estimated via *spm_coreg.m* from the Statistical Parametric Mapping (SPM; https://www.fil.ion.ucl.ac.uk/spm/) and scaled them from 0 to 255. Finally, image intensities were scaled from 0 to 255.

The 2D images used by DeepBrainNet for prediction were the 80 slices centered at the z = 0 plane in MNI coordinates of the normalized T1w. Note that our chosen brain age prediction (i.e., DeepBrainNet) method does not rely on the whole-brain MRI. This is at least convenient for axial clinical-grade MRIs, since they often lack their topmost and bottommost slices.

### Quality control of the preprocessed MRIs

Preprocessed MRI images were submitted to a careful quality control (QC) procedure. Since the study included a large number of MRIs, only a subset of the preprocessed MRIs that were likely to have bad quality were visually inspected. The selection of this subset of MRIs was carried out as follows. We calculated the normalized mutual information (NMI)^40^ between the preprocessed MRIs and the 1-mm isotropic voxel FSL skull-stripped T1w template. We then plotted the histogram of the NMIs and visually defined a threshold based on those values appearing to be significantly below the main unimodal distribution. We inspected all images below this threshold and those above it until they had no visible preprocessing errors. Since the goal is to demonstrate feasibility of the brain age estimation for clinical images, which have generally less quality than those intended for research purposes, we were lenient regarding the consideration of what a “processing error” was. We only removed preprocessed MRIs that were indisputably unrecognizable due to motion, the brain extraction did not remove significant non-brain tissues, or the normalization performed poorly. In addition, we discarded images with significant structural abnormalities (e.g., tumors, deformations, very large ventricles, tissue loss).

### Data subdivisions

**Figure 4:**
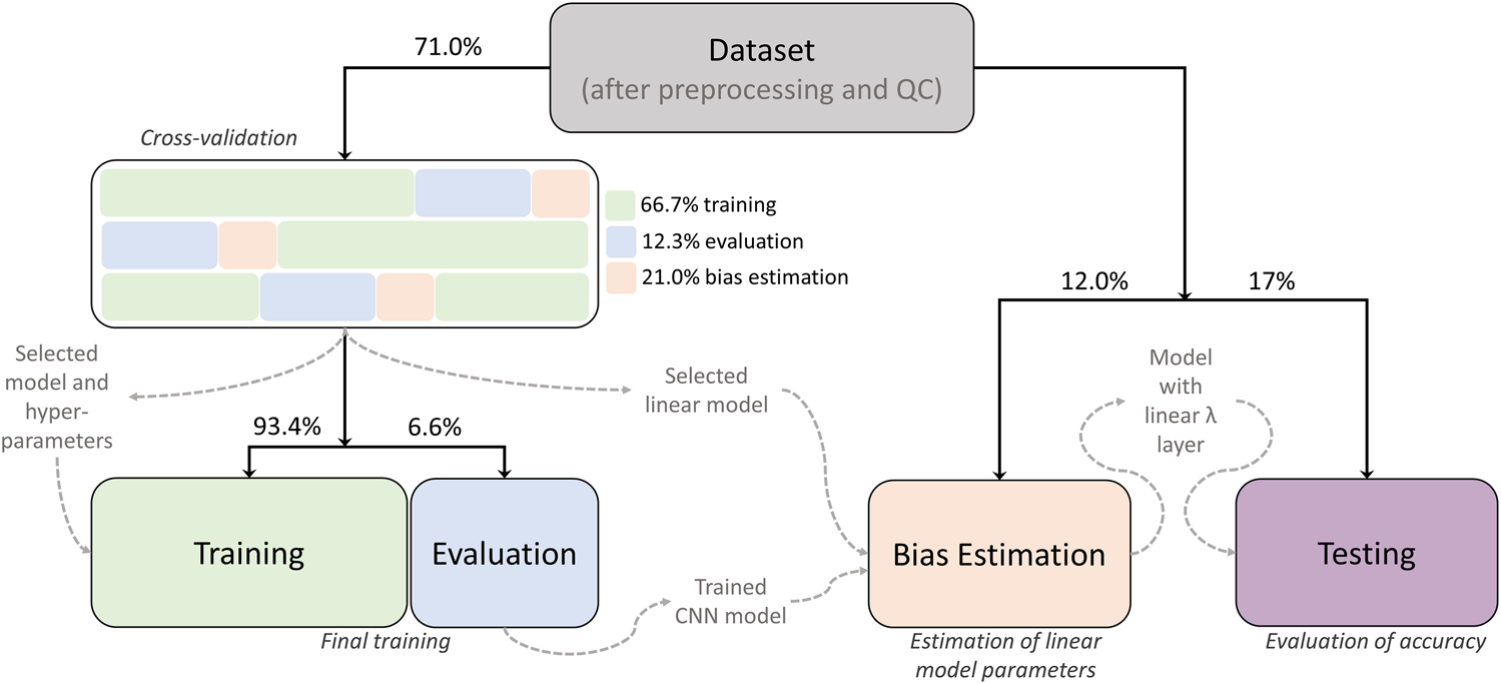
Flowchart of the definition of the data domains and the different stages of training and evaluation. After selecting the whole brain MRIs, preprocessing, and performing QC, the final dataset was split for training the CNN model (training set), estimating the parameters of the bias model in a different independent sample (the bias estimation set) and testing accuracy (external validation in the held-out testing set) and reliability. In more detail, three-fold cross-validation was used on the training set to determine the optimal combination of CNN model, tune hyperparameters and select a bias model. The final training was performed on the training set using the optimal configuration. The trained CNN model was then used to predict the bias-uncorrected brain ages that were used to estimate the parameters of the bias model in the independent bias estimation set. Finally, the bias model was added on top of the CNN for deployment and tested in the held-out testing set.

Figure 4 depicts how the dataset was split for training and evaluations. Splits were done using MATLAB *cvpartition.m* at the participant level, i.e., if a participant belonged to a certain data proportion or subdivision, all the slices of all the modalities of that participant belonged in that proportion or subdivision. We split all participants into three main independent subdivisions: 71% for training (n = 1109 participants, 4470 MRIs), 12% (n = 186 participants, 841 MRIs) for characterizing and correcting the “regression dilution” bias (i.e., the bias estimation set), and 17% (n = 264 participants, 1514 MRIs) held-out for external validation. These very specific proportions were the consequence of the distribution of modalities in the whole sample, as we explain here. First, there were three modalities (i.e., T1wFLAIRs, T2wGRE and IRs) that had too few samples to be part of the training or bias estimation sets. Thus, we decided that they could only be part of the sample used to test the predictions, allowing to test the accuracy in modalities that were not used to training the model. Most MRIs acquired using the “Signa HDxt” and “Skyra” scanners were in the testing set. Thus, we moved any remaining participant that had at least one MRI acquired with any of these two scanner models to the testing set as well, which also enabled testing the accuracy in scanner models that were not used for training. These three modalities and two scanner models were only in 17% of the participants, hence this proportion for the testing set. The training set was selected to be 65% of the whole sample for the actual training, plus its 10%, to monitor accuracy and overfitting during the training process (as we explain in the next section), hence the above-mentioned 71%. The remaining 12% of the sample was used to determine the bias correction. Therefore, the training and bias estimation sets had only MRIs among five different modalities, whereas the testing set had MRIs among the seven modalities.

In addition, the training data were split into three folds for cross-validation of the models and hyperparameters. Following the same strategy previously described for the whole dataset, at each of the three iterations of cross-validation, two folds (i.e., 66.7% of the training data) were used for training and the remaining proportion was further subdivided into 63% for correcting the bias and 37% for evaluation (and to define early stopping) during training (21% and 12.3% of the whole training data, respectively). The number of participants and MRIs for each split shown in Fig. 4 are shown in Tables S2–S15 of the Supplemental Materials.

When stratifying, we aimed to preserve the distribution of the occurrence of the values of the Cartesian product of sex, modality, scanner model and a binned chronological age across subdivisions. Given the large number of unique values of this Cartesian product, chronological age was binned in intervals of 20 years. With the training and evaluation sets having the same distribution of these variables, we guarantee that the model does not learn to amend these domain mismatches instead of maximizing accuracy. Here, the domain mismatches are the so-called “target” or “manifestation” shift when there is a mismatch in chronological age, the so-called “covariate” or “population” shift when there is mismatch in sex, the so-called “concept” or “annotation” shift when there is a mismatch in the modalities and/or scanner models^41^. Nevertheless, we considered different weighting approaches during training, based on age, sex, or combined, as described below in section “Re-training of the DeepBrainNet models”. On the other hand, given the way we created the testing sample, we could not guarantee that the variables distributed similarly between training and testing sets. While this did not bias learning, it could have affected the generalization error. Figures S1–S4 of the Supplementary Materials show the distribution of chronological age, sex, modality and scanner model. We used pairwise two-sample Kolmogorov–Smirnov to test for differences in the distributions of the chronological age among splits, and pairwise χ^2^ test for sex, modality and scanner model. As expected, after correcting for multiple comparisons (across splits in each row of the figures) using Bonferroni correction, the distribution of chronological age was only significantly different between the training and bias estimation sets (p = 0.0068). This does not bias training but rather may affect bias model selection. That is why we considered not only OLS but also WLS, weighting by modality or scanner model, when fitting the bias models. As expected, the distribution of scanner models in the testing set was significantly different from that of the training, evaluation and bias estimation sets (2.7e−200, 1.7e−54 and 1.06e−30, respectively). The rest of the pairwise comparisons for chonological age, sex, modalities, or scanner models were not statistically significant, including among the cross-validation splits. Finally, to ensure reproducibility, the samples were generated with a fixed seed at the beginning of the study.

### Re-training of the DeepBrainNet models

We used Keras 2.11 with the mean squared error (MSE) as the loss function, the mean absolute error (MAE) as the accuracy metric and the Adam optimizer^42^. To tune model hyperparameters, we cross-validated across a grid defined by the Cartesian product of the models (InceptionResnetV2 and VGG16) and the following values of the hyperparameters: learning rate = [7e−6, 7e−5, 7e−4], the batch size = [80, 160, 240, 320, 400] and a variable specifying how weights of importance of the observations were applied during training to avoid the algorithm to learn to predict brain ages more accurately for certain classes more frequent in the training data. We tried no weights, weights based on the inverse of the number of MRIs a participant had, and weights based on the inverse of the number of MRIs and prevalence of chronological age and sex, multiplied. The training data was shuffled at the slice level so a batch could include slices from several subjects.

For each cross-validation iteration and grid cell, we trained the original CNN model on the training subset, evaluated its performance using MAE on the validation subset, corrected the linear bias using the bias estimation subset, and calculated a bias-corrected MAE again in the validation subset. This was done as follows.

We loaded the corresponding original DeepBrainNet model and, at first, set the weights of the fully connected and output layers as trainable, while the weights of the rest of the network were frozen. We trained this model using a maximum of 20 epochs. We then unfroze all the layers of the model and re-trained it with another maximum of 20 epochs. Early stopping was applied during training if there was no improvement of the loss function during the last three consecutive epochs (patience = 3) or if the MAE of the training data was lower than that of the validation data (patience = 0) to avoid overfitting. During training, we adjusted the learning rate after each batch using $\text{learning rate}\,\left(\text{batch}\right)=\text{initial learning rate }/\left(1 +\text{ batch}\times \text{decay}\right)$, decay = 3e−7. After the end of the training, we used the trained model to predict the brain age of each slice in the bias estimation subset of the cross-validation iteration and computed the median within subjects to obtain the individual whole-brain brain ages.

We then fitted the bias model with generic form $brain age={f}_{{\varvec{\beta}}}\left(chronological\, age\right)+error$, where ${f}_{{\varvec{\beta}}}\left(chronological\, age\right)$ is a function of $chronological \,age$ that depends on a set of regression coefficients contained in the vector ${\varvec{\beta}}$. We proposed the following forms, written in Wilkinson notation for the sake of simplicity:

1. ${f}_{{\varvec{\beta}}}\left(chronological\, age\right)=chronological\, age$, where ${\varvec{\beta}}$ contains 2 elements.
2. ${f}_{{\varvec{\beta}}}\left(chronological\, age\right)=chronological\, age\times modality$, where ${\varvec{\beta}}$ contains 10 elements.
3. ${f}_{{\varvec{\beta}}}\left(chronological \,age\right)=chronological \,age\times scanner$, where ${\varvec{\beta}}$ contains 16 elements.

We then added an additional layer to the model’s architecture (using the Keras’ “Lambda” layer) that takes the modality and/or the scanner model (if bias model requires it), and outputs a bias-corrected brain age according to the formula described in the next section. We shall call this new model the “corrected model”. Finally, we predicted the brain age in the evaluation subset of the cross-validation iteration using the trained uncorrected model to obtain a “bias-uncorrected MAE” for that cross-validation iteration and grid cell, and the corresponding corrected models to obtain a “bias-corrected MAEs” for each bias model for that cross-validation iteration and grid cell.

We selected the models and training parameters that yielded the lowest MAE averaged across folds. We used the bias-uncorrected MAE to select the CNN and set of training hyper-parameters. We then used the bias-corrected MAEs of the selected CNN/hyper-parameter combination to select the bias correction formula. Using this final combination, we re-trained the original CNN model as described above using 93.4% of the participants of the whole training set, keeping only 6.6%  of the whole training set to monitor early stopping. We then used the held-out bias estimation set to correct the linear bias using the selected bias model. Finally, we evaluate the generalization error and internal consistency in the held-out testing set as described in the next section.

### Correcting the bias in brain ages

The use of age as a covariate in group-level analyses commonly seen in the literature invites the following correction of the brain age to remove the bias:

$$Corrected\, brain\, age=chronological \,age+brain\, age-{f}_{\widehat{{\varvec{\beta}}}}\left(chronological \,age\right).$$

However, even though using ${f}_{\widehat{{\varvec{\beta}}}}\left(chronological\, age\right)$ as a covariate in group regressions of brain-PAD is appropriate to account for unwanted age-related variance, the presence of chronological age-dependent terms in this equation misleads the accuracy of the unbiased brain ages obtained using this equation. For example, for model 1 in the previous section, i.e., ${f}_{\widehat{{\varvec{\beta}}}}\left(chronological \,age\right)=chronological \,age$ in Wilkinson notation, Butler et al. theoretically and empirically demonstrated that the correlation between the chronological ages and the bias-corrected predicted brain ages obtained via this equation, ${r}_{corrected \,brain\, age,\,age}$, is inflated and never below ~ 0.87, even if there is no relationship between the MRIs and age at all^28^. And this lower bound is when the sample used to estimate the coefficients is not the same as the sample for which brain age is corrected. If the same sample is used for both, the situation worsens, as ${r}_{corrected\, brain\, age,\,age}$ ≥ ~ 0.9177^28^. Consequently, the MAE of the bias-corrected brain ages is also spuriously lower than the MAE of the bias-uncorrected ones.

We thus propose the following chronological age-independent correction:

1 $$Corrected\, brain \,age={f}_{\widehat{{\varvec{\beta}}}}^{-1}\left(brain \,age\right).$$

Note that Eq. (1) is a generalization of the correction proposed by Cole et al.^4^ for model 1 in the previous section, i.e., $corrected \,brain\, age=\frac{brain age-{\widehat{\beta }}_{intercept}}{{\widehat{\beta }}_{linear}}$. In this particular case, there is no risk of overestimation of the accuracy since ${r}_{corrected\, brain\, age,\,age}$ is identical to ${r}_{brain \,age,\,age}$, whereas the variance of the bias-corrected brain ages is $1/{\widehat{\beta}^2_{linear}}$ times higher than the variance of the bias-uncorrected brain ages. Corrections based on models 2 or 3 are equivalent to that of model 1, but using the coefficients corresponding to each individual’s modality or scanner model, respectively. When correcting a brain age from an MRI with a modality or scanner model not present in the bias estimation set (e.g., the T2wGRE modality or Signa HDxt scanner), the average across modalities or scanner models, respectively, of the parameters (intercepts and slopes) was used. Note that the correction does not depend on whether OLS or WLS is used to fit the bias model.

### Evaluation of the accuracy in the testing dataset (generalization error)

We reported the accuracy of the predictions using the sample MAE, the correlation between the predicted brain age and the chronological age, and the coefficient of determination (R^2^) of the $brain\, age=chronological\, age+error$ linear model (i.e., $slope=1$ and $intercept=0$). That is, with $\langle x\rangle$ being the average of $x$:

$$\begin{array}{l}{R}^{2}=1-\left(\frac{RSS}{TSS}\right),\\ RSS={\sum }_{i}{\left({brain\, age}_{i}-{chronological \,age}_{i}\right)}^{2},\\ TSS={\sum }_{i}{\left({brain\, age}_{i}-\langle brain \,age\rangle \right)}^{2}.\end{array}$$

A perfect fit yields R^2^ = 1. Also, with this constrained definition, R^2^ can have a negative value when the model $brain\, age=chronological\, age+error$ poorly follows the trend of the data.

We adopted a non-parametric approach to calculate these measures of accuracy to deal with possible inflation due to repeated observations. We calculated these measures separately for each modality using a randomly selected repetition per subject. Since this method yields a different result for each run, we used bootstrapping to calculate the accuracy measures and took the average. Bootstrapping also allowed us to estimate the 95% confidence intervals (CIs). We used 10,000 × N_r_ bootstraps to account for the fact a random image among a maximum of N_r_ repeated measures is drawn for each subject and modality. When reporting the accuracy measures for the total sample, i.e., pooling from modalities and repetitions, we used 10,000 × N_r_ × N_m_ bootstraps, where N_m_ is the number of modalities.

To test part of our first hypothesis, that involves comparing predictive accuracies, we fitted the linear mixed model “*|brain-PAD|*~ *modality* + *subject* + (1*|subject*)”, where *|x|* takes the absolute value of *x*, *modality* is a categorical variable with a level for each modality, including the actual MPRAGE. Note that this is a mixed effects repeated measures ANOVA with missing values in long format, i.e., $|{brain\, PAD}_{i,r,m} |={s}_{i}+{\beta }_{m}+{\eta }_{i}+{e}_{i,r,m}$ where, ${s}_{i}$ is the $i{\text{th}}$’s subject fixed effect, ${\beta }_{m}$ is the fixed effect of the $m{\text{th}}$ modality, ${\eta }_{i}$ is $i{\text{th}}$’s subject the random effect and ${e}_{i,r,m}$ is a random error for the $i{\text{th}}$ subject $r{\text{th}}$ repetition and $m{\text{th}}$ modality. The fit was done by maximizing the Restricted Maximum Likelihood using the ‘Quasi-Newton’ optimizer and modeling the covariance matrix using a full Cholesky parameterization. By modeling a non-diagonal covariance matrix and adding a random effect in the intercept across subjects, we are accounting for possible correlations between observations due to the use of repeated measures for some subjects (between and within modalities). This should avoid inflation of Type I errors when testing the significance of certain contrasts in the model. We then tested the within-subject comparison of the *|brain-PAD|* between the $m{\text{th}}$ synthetic modality and the actual MPRAGE by evaluating and testing the contrast that compared their corresponding coefficients in the model, i.e., ${\beta }_{m}-{\beta }_{MPRAGE}$, ${\beta }_{m}$ is the coefficient of the fixed effect of the $m{\text{th}}$ modality.

Finally, note that this linear mixed model also provides an estimation of the absolute value of the brain-PAD of each subject’s modality given by ${s}_{i}+{\beta }_{m}$, where ${s}_{i}$ is the coefficient of the fixed effect of the $ith$ subject. Thus, we can also report a “population” estimate of the MAE. To that end, we evaluated the contrast ${\widehat{MAE}}_{m}={\langle {s}_{i}\rangle }_{i}+{\beta }_{m}$, where ${\langle \rangle }_{i}$ denotes averaging across $i$, for an estimation of the MAE for each modality, and the contrast ${\langle {\widehat{MAE}}_{m}\rangle }_{m}$ for an estimation of the total MAE.

### Reliability of brain age predictions across modalities and repetitions

To test our second hypothesis, we also evaluated the intra-subject reliability of the predictions, specifically, we evaluated Cronbach’s alpha on the brain-PAD. The Cronbach’s alpha is a measure of internal consistency^31^. The ranges α ≥ 0.9 and 0.8 ≤ α < 0.9 denote an excellent and acceptable reliability, respectively, whereas lower values denote questionable, poor or even unacceptable reliability. Very high values of α ≥ 0.95 denote redundancy (which is desirable in our case). For the calculation of the Cronbach’s alpha, we reorganized the values into a matrix, $X$, of number of participants by modality/repetition pairs (the items). We considered all possible 6 × 4 = 24 modality/repetition pairs (i.e., the MPRAGE and the synthetic MRIs for all five modalities, and the maximum of four repetitions). We then dropped those participants (rows) having less than 3 items, and those items having more than 95% of missing values. Cronbach’s alpha was calculated using the following formula^43^:

$$Cronbach=\frac{number \,of\, items}{number\, of\, items-1}\left[1-\frac{trace\left(C\right)}{\sum_{ij}{C}_{ij}}\right],$$

where $C$ is the covariance matrix of $X$. To handle the remaining missing values, C was calculated using pairwise elimination. The lower and upper bounds of a CI (e.g., 95%) for the Cronbach’s alpha were given by:

$$\begin{array}{l}Lower=1-\left(1-cronbach\right){F}^{-1}\left(\alpha /2,{df}_{1},{df}_{2}\right)\\ Upper=1-\left(1-cronbach\right){F}^{-1}\left(1-\alpha /2,{df}_{1},{df}_{2}\right)\end{array},$$

where ${F}^{-1}()$ is the inverse of the complementary cumulative distribution function (or inverse survival function) for the F-distribution, $\alpha =1- CI/100$, $df1=number\, of\, observations- 1$, and $df2= df1\times number\, of\, items$.

### Supplementary Information

Supplementary Information.

## Data Availability

The datasets generated and/or analyzed during the current study are not publicly available because the related project has not concluded, but are available from the corresponding author on reasonable request.
