# Supplementary material for: Toward MR protocol-agnostic, unbiased brain age predicted from clinical-grade MRIs
Source: Sci Rep. 2023 Nov 10;13:19570. doi: 10.1038/s41598-023-47021-y (PMC10638359; doi:10.1038/s41598-023-47021-y)
Supplement: Supplementary file 1 — Supplementary Information. [file 41598_2023_47021_MOESM1_ESM.docx]

Supplemental Materials

Toward MR protocol-agnostic, unbiased brain age predicted from clinical-grade MRIs.

Pedro A. Valdes-Hernandez^1,2,3*^, Chavier Laffitte Nodarse^1,2,3^, Julio A. Peraza^4^, James H. Cole^5^,^6^, Yenisel Cruz-Almeida^1,2,3,7^

1. Department of Community Dentistry and Behavioral Science, University of Florida, USA
2. Pain Research and Intervention Center of Excellence, University of Florida, USA
3. Center for Cognitive Aging and Memory, McKnight Brain Institute, University of Florida, USA
4. Department of Physics, Florida International University, USA
5. Centre for Medical Image Computing, Department of Computer Science, University College London, UK
6. Dementia Research Centre, Queen Square Institute of Neurology, University College London, UK
7. Department of Neuroscience, College of Medicine, University of Florida, USA

***Corresponding author:**

Pedro A. Valdes-Hernandez, PhD

1329 SW 16th Street, Ste. 5180, Gainesville, FL 32610

Email: pvaldeshernandez@dental.ufl.edu

Table S1. Summary of some of the parameters of the clinical MRI protocols

| Scanner model  Manufacturer/  Field Strength | *Modality* | *Voxel size*  *Mean (SD)*  *[x, y, z]* | *Voxel size*  *Min.-Max.*  *[x, y, z]* | *TE [ms.]*  *Mean (SD)* | *TR [ms.]*  *Mean (SD)* | *Axial/*  *Coronal/ Sagittal* |
| --- | --- | --- | --- | --- | --- | --- |
| **Aera**  **Siemens**  **1.5 T** | **MPRAGE** | [0.9 (0.1), 0.8 (0.2), 0.9 (0.1)] | [0.5 - 1.0, 0.4 - 1.0, 0.9 - 1.2] | 2.5  (0.2) | 1847.6  (314.0) | 21/0/136 |
|  | **T1w** | [0.7 (0.1), 0.7 (0.1), 4.0 (0.2)] | [0.3 - 1.0, 0.3 - 1.0, 1.0 - 4.0] | 7.3  (2.4) | 346.4  (130.3) | 184/0/77 |
|  | **T2w** | [0.5 (0.2), 0.5 (0.2), 3.9 (0.4)] | [0.3 - 1.0, 0.3 - 1.0, 0.8 - 4.0] | 92.2 (19.7) | 5592.9  (812.9) | 196/195/8 |
|  | **T1wFLAIR** | [0.9 (0.0), 0.9 (0.0), 3.5 (0.7)] | [0.9 - 0.9, 0.9 - 0.9, 3.0 - 4.0] | 7.2  (0.0) | 2930.0  (424.3) | 1/0/1 |
|  | **T2wFLAIR** | [0.7 (0.1), 0.7 (0.1), 4.0 (0.3)] | [0.4 - 0.9, 0.4 - 0.9, 0.9 - 5.0] | 85.6  (25.3) | 8963.5  (371.0) | 227/0/4 |
|  | **IR** | [1.0 (0.1), 0.9 (0.1), 0.9 (0.1)] | [0.9 - 1.0, 0.9 - 1.0, 0.9 - 1.0] | 344.8  (19.5) | 5000.0  (0.0) | 1/0/3 |
| **Avanto**  **Siemens**  **1.5 T** | **MPRAGE** | [0.9 (0.1), 0.9 (0.1), 1.0 (0.1)] | [0.5 - 1.0, 0.5 - 1.0, 0.9 - 1.2] | 3.3  (0.6) | 1665.2  (205.4) | 61/0/147 |
|  | **T1w** | [0.5 (0.1), 0.5 (0.1), 4.0 (0.2)] | [0.4 - 1.0, 0.4 - 1.0, 1.0 - 5.0] | 9.5  (2.0) | 591.2  (79.8) | 301/0/203 |
|  | **T2w** | [0.7 (0.1), 0.7 (0.1), 3.9 (0.4)] | [0.3 - 1.3, 0.3 - 1.3, 1.0 - 5.0] | 105.1  (26.9) | 5845.9  (1513.3) | 309/239/17 |
|  | **T1wFLAIR** | [0.7 (0.2), 0.7 (0.2), 3.2 (0.5)] | [0.4 - 0.9, 0.4 - 0.9, 3.0 - 4.0] | 14.1  (18.1) | 2457.5  (226.4) | 7/0/1 |
|  | **T2wFLAIR** | [0.6 (0.2), 0.6 (0.2), 3.8 (0.8)] | [0.4 - 1.0, 0.4 - 1.0, 1.0 - 5.0] | 109.5  (67.5) | 8747.5  (855.2) | 110/0/8 |
|  | **IR** | [1.0 (0.1), 1.1 (0.1), 1.1 (0.1)] | [1.0 - 1.3, 1.0 - 1.3, 1.0 - 1.3] | 369.7  (26.0) | 5833.3  (389.2) | 0/0/13 |
| **Prisma**  **Siemens**  **3.0 T** | **MPRAGE** | [0.9 (0.1), 0.8 (0.2), 0.9 (0.0)] | [0.4 - 1.0, 0.4 - 1.0, 0.9 - 1.0] | 2.3  (0.1) | 1748.5  (223.7) | 37/0/132 |
|  | **T1w** | [0.6 (0.2), 1.0 (1.3), 3.9 (0.4)] | [0.3 - 1.0, 0.3 - 5.0, 0.5 - 4.0] | 6.9  (4.5) | 728.2  (578.2) | 149/27/58 |
|  | **T2w** | [0.6 (0.1), 0.6 (0.1), 3.9 (0.5)] | [0.2 - 1.0, 0.2 - 0.7, 0.9 - 4.0] | 110.4  (47.1) | 5131.1  (571.9) | 175/107/15 |
|  | **T1wFLAIR** | [0.6 (0.0), 0.6 (0.0), 3.0 (0.0)] | [0.6 - 0.6, 0.6 - 0.6, 3.0 - 3.0] | 11.0  (1.4) | 1469.0  (1033.8) | 2/0/0 |
|  | **T2wFLAIR** | [0.7 (0.1), 0.6 (0.1), 3.8 (0.8)] | [0.3 - 1.1, 0.3 - 1.1, 0.9 - 4.0] | 98.6  (72.1) | 8753.3  (941.2) | 211/0/15 |
|  | **IR** | [1.0 (0.1), 0.6 (0.3), 1.0 (0.1)] | [0.9 - 1.4, 0.4 - 1.4, 0.9 - 1.4] | 346.9  (62.7) | 6027.8  (1264.6) | 0/0/36 |
| **Sola**  **Siemens**  **1.5 T** | **MPRAGE** | [0.9 (0.0), 0.8 (0.2), 0.9 (0.0)] | [0.9 - 0.9, 0.4 - 0.9, 0.9 - 0.9] | 2.6  (0.2) | 2134.5  (195.5) | 1/0/48 |
|  | **T1w** | [0.8 (0.0), 0.8 (0.0), 4.0 (0.2)] | [0.6 - 0.9, 0.6 - 0.9, 3.0 - 4.0] | 12.6  (1.0) | 668.7  (96.9) | 32/0/0 |
|  | **T2w** | [0.4 (0.2), 0.4 (0.2), 4.0 (0.1)] | [0.3 - 0.8, 0.3 - 0.8, 3.0 - 4.0] | 84.2  (2.4) | 5035.7  (546.7) | 46/2/0 |
|  | **T2wFLAIR** | [0.7 (0.1), 0.7 (0.1), 3.9 (0.4)] | [0.6 - 0.9, 0.6 - 0.9, 0.9 - 4.0] | 93.5  (43.5) | 8958.3  (288.7) | 47/0/1 |
|  | **IR** | [0.9 (0.0), 0.9 (0.0), 0.9 (0.0)] | [0.9 - 0.9, 0.9 - 0.9, 0.9 - 0.9] | 386.0  (0.0) | 7000.0  (0.0) | 0/0/1 |
| **Signa HDxt**  **GE Medical Systems**  **1.5 T** | **MPRAGE** | [0.9 (0.1), 0.9 (0.1), 1.0 (0.1)] | [0.5 - 1.2, 0.5 - 1.1, 0.9 - 1.2] | 2.8  (0.3) | 8.8  (0.9) | 15/0/74 |
|  | **T1w** | [0.7 (0.2), 0.7 (0.2), 4.0 (0.2)] | [0.4 - 1.1, 0.4 - 1.1, 4.0 - 5.0] | 21.8  (5.6) | 1789.6  (839.3) | 95/0/62 |
|  | **T2w** | [0.4 (0.0), 0.4 (0.0), 4.0 (0.1)] | [0.4 - 0.5, 0.4 - 0.5, 3.0 - 5.0] | 100.9  (5.0) | 5367.9  (1191.1) | 128/139/3 |
|  | **T1wFLAIR** | [0.5 (0.2), 0.5 (0.2), 4.0 (0.0)] | [0.4 - 0.9, 0.4 - 0.9, 4.0 - 4.0] | 24.9  (1.1) | 2427.1  (488.4) | 53/0/20 |
|  | **T2wFLAIR** | [0.4 (0.0), 0.4 (0.0), 4.0 (0.2)] | [0.4 - 0.5, 0.4 - 0.5, 4.0 - 5.0] | 124.6  (2.0) | 9061.3  (254.6) | 150/0/1 |
|  | **T2wGRE** | [0.8 (0.1), 0.8 (0.1), 4.0 (0.2)] | [0.4 - 1.1, 0.4 - 1.1, 4.0 - 5.0] | 13.2  (0.4) | 561.9  (42.5) | 154/0/0 |
| **Skyra**  **Siemens**  **3.0 T** | **MPRAGE** | [0.9 (0.1), 0.7 (0.2), 0.9 (0.0)] | [0.5 - 1.0, 0.4 - 1.0, 0.9 - 1.0] | 2.3  (0.1) | 1803.8  (237.9) | 3/0/28 |
|  | **T1w** | [0.7 (0.1), 0.7 (0.1), 3.7 (0.9)] | [0.6 - 0.9, 0.6 - 0.9, 0.9 - 4.0] | 7.9  (4.4) | 468.2  (196.4) | 21/0/10 |
|  | **T2w** | [0.5 (0.1), 0.5 (0.1), 4.1 (0.4)] | [0.3 - 0.7, 0.3 - 0.7, 3.0 - 5.0] | 105.4  (9.5) | 4997.3  (937.7) | 29/15/0 |
|  | **T1wFLAIR** | [0.6 (0.0), 0.6 (0.0), 3.0 (0.0)] | [0.6 - 0.6, 0.6 - 0.6, 3.0 - 3.0] | 9.6  (0.0) | 2040.0  (0.0) | 1/0/0 |
|  | **T2wFLAIR** | [0.7 (0.1), 0.7 (0.1), 3.4 (1.2)] | [0.6 - 1.0, 0.5 - 1.0, 0.9 - 4.0] | 144.5  (122.8) | 8200.0  (1620.4) | 32/0/6 |
|  | **IR** | [1.0 (0.1), 0.9 (0.2), 1.0 (0.1)] | [0.9 - 1.4, 0.5 - 1.0, 0.9 - 1.4] | 355.1  (35.1) | 6166.7  (1291.0) | 0/0/17 |
| **Titan3T**  **TOSHIBA**  **1.5 T** | **MPRAGE** | [0.6 (0.2), 0.8 (0.3), 1.1 (0.1)] | [0.5 - 1.2, 0.5 - 1.2, 1.0 - 1.2] | 3.2  (0.6) | 6.7  (1.8) | 13/0/25 |
|  | **T1w** | [0.3 (0.0), 0.3 (0.0), 3.9 (0.3)] | [0.3 - 0.4, 0.3 - 0.4, 3.0 - 4.0] | 10.7  (9.2) | 837.0  (537.9) | 81/0/34 |
|  | **T2w** | [0.4 (0.0), 0.4 (0.0), 4.0 (0.0)] | [0.3 - 0.5, 0.3 - 0.5, 4.0 - 4.0] | 108.0  (0.0) | 6920.5  (1296.6) | 65/58/1 |
|  | **T2wFLAIR** | [0.7 (0.1), 0.7 (0.1), 4.0 (0.0)] | [0.4 - 0.9, 0.4 - 0.9, 4.0 - 4.0] | 104.2  (8.2) | 9841.5  (367.5) | 87/0/0 |
| **Verio**  **Siemens**  **3.0 T** | **MPRAGE** | [0.9 (0.1), 0.8 (0.2), 0.9 (0.1)] | [0.5 - 1.2, 0.4 - 1.0, 0.9 - 1.2] | 3.3  (2.0) | 1938.1  (328.4) | 40/0/159 |
|  | **T1w** | [0.6 (0.1), 0.6 (0.1), 4.0 (0.0)] | [0.3 - 0.8, 0.3 - 0.8, 4.0 - 4.0] | 6.0  (4.3) | 409.8  (192.7) | 264/1/170 |
|  | **T2w** | [0.5 (0.2), 0.5 (0.2), 3.8 (0.5)] | [0.3 - 1.0, 0.3 - 0.9, 1.0 - 4.0] | 113.0  (31.1) | 5104.4  (1455.8) | 250/216/8 |
|  | **T1wFLAIR** | [0.7 (0.1), 0.7 (0.1), 3.5 (0.6)] | [0.7 - 0.9, 0.7 - 0.9, 3.0 - 4.0] | 20.5  (18.3) | 2690.0  (474.6) | 3/0/1 |
|  | **T2wFLAIR** | [0.6 (0.2), 0.5 (0.1), 3.6 (1.1)] | [0.4 - 1.0, 0.4 - 0.9, 0.9 - 5.0] | 149.5  (104.7) | 8388.9  (1378.0) | 94/0/16 |
|  | **IR** | [1.0 (0.1), 0.5 (0.1), 1.0 (0.1)] | [0.9 - 1.2, 0.4 - 0.9, 0.9 - 1.2] | 369.8 (33.3) | 6076.9  (1189.1) | 0/0/27 |

Note. The last column indicates the number of images for the corresponding scanner and modality for the each of the three orientations. MPRAGE: Magnetization-prepared rapid gradient-echo. T1w: T1-weighted. T2w: T2-weighted. FLAIR: Fluid attenuated inversion recovery. GRE: Gradient echo. It is worth noting that the slice orientation of some MPRAGEs is not sagittal as expected, even though, for some, their protocol name and/or sequence name says so. For these cases, the DICOM’s “ImageOrientationPatient” attribute indicates that the slice orientation was modified to axial, exposing the arbitrary nature of clinical MR protocols.

Table S2-S15 below show the distribution of MRIs that passed QC among modalities and scanners. The first section shows the number of MRIs per modality and scanner. The second section shows the number of subjects having 1, 2, 3 or 4 repetitions of each modality. Note that the number of original MPRAGEs and synthetic MPRAGEs may not coincide. This is because some original MPRAGEs passed the QC after preprocessing while its synthetic counterpart did not, and vice versa. Also, for each column, the total of MPRAGEs (either original or synthetic) among the training, evaluation, linear correction and testing sets (i.e., summing the corresponding cells of the first or second row across Tables S3-6) is not equal to the corresponding number of MPRAGEs in Table 1 of the main text of the paper. That is because that table reports the total of original MPRAGEs that were used in the study, irrespective of whether they were used as original only, synthetic only, or both. A similar situation happens with the totals (last row of the tables), because here we report the total of images used (including originals and synthetic MPRAGEs) and Table 1 only reports the total actual MRIs used in the study.

Table S2. Training and Evaluation Set (used for cross-validation training).

| **Modality** | **Scanner** | | | | | | | |  | **Number of repetitions** | | | |
| --- | --- | --- | --- | --- | --- | --- | --- | --- | --- | --- | --- | --- | --- |
|  | **Aera** | **Avanto** | **Prisma** | **Sola** | **Signa**  **HDxt** | **Skyra** | **Titan**  **3T** | **Verio** | **TOTAL** | **1** | **2** | **3** | **4** |
| **MPRAGE** | 118 | 158 | 121 | 42 | 0 | 0 | 26 | 149 | 614 | 602 | 6 | 0 | 0 |
| **Synthetic MPRAGEs** | | | | | | | | | | | | | |
| **MPRAGE** | 121 | 156 | 123 | 41 | 0 | 0 | 29 | 152 | 622 | 610 | 6 | 0 | 0 |
| **2D T1w** | 215 | 425 | 197 | 25 | 0 | 0 | 100 | 371 | 1,333 | 503 | 399 | 8 | 2 |
| **2D T2w** | 324 | 469 | 218 | 42 | 0 | 0 | 107 | 382 | 1,542 | 440 | 517 | 20 | 2 |
| **2D T1wFLAIR** | 0 | 0 | 0 | 0 | 0 | 0 | 0 | 0 | 0 | 0 | 0 | 0 | 0 |
| **2D T2wFLAIR** | 193 | 88 | 180 | 41 | 0 | 0 | 76 | 81 | 659 | 637 | 11 | 0 | 0 |
| **2D T2wGRE** | 0 | 0 | 0 | 0 | 0 | 0 | 0 | 0 | 0 | 0 | 0 | 0 | 0 |
| **2D IR** | 0 | 0 | 0 | 0 | 0 | 0 | 0 | 0 | 0 | 0 | 0 | 0 | 0 |
| **TOTAL** | 971 | 1,296 | 839 | 191 | 0 | 0 | 338 | 1135 | 4,770 | 2,792 | 939 | 28 | 4 |

Table S3. Training (used for final training the selected CNN model and hyper-parameter configuration).

| **Modality** | **Scanner** | | | | | | | |  | **Number of repetitions** | | | |
| --- | --- | --- | --- | --- | --- | --- | --- | --- | --- | --- | --- | --- | --- |
|  | **Aera** | **Avanto** | **Prisma** | **Sola** | **Signa**  **HDxt** | **Skyra** | **Titan**  **3T** | **Verio** | **TOTAL** | **1** | **2** | **3** | **4** |
| **MPRAGE** | 112 | 144 | 113 | 40 | 0 | 0 | 25 | 139 | 573 | 561 | 6 | 0 | 0 |
| **Synthetic MPRAGEs** | | | | | | | | | | | | | |
| **MPRAGE** | 114 | 142 | 115 | 39 | 0 | 0 | 28 | 142 | 580 | 568 | 6 | 0 | 0 |
| **2D T1w** | 197 | 392 | 187 | 23 | 0 | 0 | 95 | 346 | 1,240 | 467 | 372 | 7 | 2 |
| **2D T2w** | 299 | 433 | 209 | 40 | 0 | 0 | 101 | 355 | 1,437 | 417 | 479 | 18 | 2 |
| **2D T1wFLAIR** | 0 | 0 | 0 | 0 | 0 | 0 | 0 | 0 | 0 | 0 | 0 | 0 | 0 |
| **2D T2wFLAIR** | 180 | 81 | 169 | 39 | 0 | 0 | 73 | 75 | 617 | 599 | 9 | 0 | 0 |
| **2D T2wGRE** | 0 | 0 | 0 | 0 | 0 | 0 | 0 | 0 | 0 | 0 | 0 | 0 | 0 |
| **2D IR** | 0 | 0 | 0 | 0 | 0 | 0 | 0 | 0 | 0 | 0 | 0 | 0 | 0 |
| **TOTAL** | 902 | 1,192 | 793 | 181 | 0 | 0 | 322 | 1,057 | 4,447 | 2,612 | 872 | 25 | 4 |

Table S4. Evaluation Set (used for monitoring overfitting during the final training of the selected CNN model and hyper-parameter configuration).

| **Modality** | **Scanner** | | | | | | | |  | **Number of repetitions** | | | |
| --- | --- | --- | --- | --- | --- | --- | --- | --- | --- | --- | --- | --- | --- |
|  | **Aera** | **Avanto** | **Prisma** | **Sola** | **Signa**  **HDxt** | **Skyra** | **Titan**  **3T** | **Verio** | **TOTAL** | **1** | **2** | **3** | **4** |
| **MPRAGE** | 6 | 14 | 8 | 2 | 0 | 0 | 1 | 10 | 41 | 41 | 0 | 0 | 0 |
| **Synthetic MPRAGEs** | | | | | | | | | | | | | |
| **MPRAGE** | 7 | 14 | 8 | 2 | 0 | 0 | 1 | 10 | 42 | 42 | 0 | 0 | 0 |
| **2D T1w** | 18 | 33 | 10 | 2 | 0 | 0 | 5 | 25 | 93 | 36 | 27 | 1 | 0 |
| **2D T2w** | 25 | 36 | 9 | 2 | 0 | 0 | 6 | 27 | 105 | 23 | 38 | 2 | 0 |
| **2D T1wFLAIR** | 0 | 0 | 0 | 0 | 0 | 0 | 0 | 0 | 0 | 0 | 0 | 0 | 0 |
| **2D T2wFLAIR** | 13 | 7 | 11 | 2 | 0 | 0 | 3 | 6 | 42 | 38 | 2 | 0 | 0 |
| **2D T2wGRE** | 0 | 0 | 0 | 0 | 0 | 0 | 0 | 0 | 0 | 0 | 0 | 0 | 0 |
| **2D IR** | 0 | 0 | 0 | 0 | 0 | 0 | 0 | 0 | 0 | 0 | 0 | 0 | 0 |
| **TOTAL** | 69 | 104 | 46 | 10 | 0 | 0 | 16 | 78 | 323 | 180 | 67 | 3 | 0 |

Table S5. Linear Correction Set (used for fitting the selected bias model).

| **Modality** | **Scanner** | | | | | | | |  | **Number of repetitions** | | | |
| --- | --- | --- | --- | --- | --- | --- | --- | --- | --- | --- | --- | --- | --- |
|  | **Aera** | **Avanto** | **Prisma** | **Sola** | **Signa**  **HDxt** | **Skyra** | **Titan**  **3T** | **Verio** | **TOTAL** | **1** | **2** | **3** | **4** |
| **MPRAGE** | 24 | 25 | 22 | 6 | 0 | 0 | 4 | 26 | 107 | 105 | 1 | 0 | 0 |
| **Synthetic MPRAGEs** | | | | | | | | | | | | | |
| **MPRAGE** | 23 | 27 | 22 | 6 | 0 | 0 | 4 | 25 | 107 | 105 | 1 | 0 | 0 |
| **2D T1w** | 43 | 72 | 31 | 6 | 0 | 0 | 15 | 60 | 227 | 95 | 64 | 0 | 1 |
| **2D T2w** | 66 | 78 | 47 | 6 | 0 | 0 | 17 | 72 | 286 | 59 | 104 | 5 | 1 |
| **2D T1wFLAIR** | 0 | 0 | 0 | 0 | 0 | 0 | 0 | 0 | 0 | 0 | 0 | 0 | 0 |
| **2D T2wFLAIR** | 34 | 20 | 31 | 6 | 0 | 0 | 11 | 12 | 114 | 112 | 1 | 0 | 0 |
| **2D T2wGRE** | 0 | 0 | 0 | 0 | 0 | 0 | 0 | 0 | 0 | 0 | 0 | 0 | 0 |
| **2D IR** | 0 | 0 | 0 | 0 | 0 | 0 | 0 | 0 | 0 | 0 | 0 | 0 | 0 |
| **TOTAL** | 190 | 222 | 153 | 30 | 0 | 0 | 51 | 195 | 841 | 476 | 171 | 5 | 2 |

Table S6. Testing Set (used for reporting the generalization error of the brain age predictions, i.e., the bias-uncorrected and bias-corrected MAE, R^2^ and correlation).

| **Modality** | **Scanner** | | | | | | | |  | **Number of repetitions** | | | |
| --- | --- | --- | --- | --- | --- | --- | --- | --- | --- | --- | --- | --- | --- |
|  | **Aera** | **Avanto** | **Prisma** | **Sola** | **Signa**  **HDxt** | **Skyra** | **Titan**  **3T** | **Verio** | **TOTAL** | **1** | **2** | **3** | **4** |
| **MPRAGE** | 4 | 11 | 21 | 1 | 87 | 30 | 0 | 12 | 166 | 160 | 3 | 0 | 0 |
| **Synthetic MPRAGEs** | | | | | | | | | | | | | |
| **MPRAGE** | 4 | 13 | 22 | 1 | 81 | 30 | 0 | 17 | 168 | 160 | 4 | 0 | 0 |
| **2D T1w** | 3 | 7 | 6 | 1 | 157 | 31 | 0 | 4 | 209 | 77 | 63 | 2 | 0 |
| **2D T2w** | 9 | 18 | 32 | 0 | 270 | 44 | 0 | 20 | 393 | 102 | 135 | 7 | 0 |
| **2D T1wFLAIR** | 2 | 8 | 2 | 0 | 73 | 1 | 0 | 4 | 90 | 50 | 20 | 0 | 0 |
| **2D T2wFLAIR** | 4 | 10 | 15 | 1 | 151 | 38 | 0 | 17 | 236 | 222 | 7 | 0 | 0 |
| **2D T2wGRE** | 4 | 13 | 36 | 1 | 0 | 17 | 0 | 27 | 98 | 34 | 32 | 0 | 0 |
| **2D IR** | 0 | 0 | 0 | 0 | 154 | 0 | 0 | 0 | 154 | 154 | 0 | 0 | 0 |
| **TOTAL** | 30 | 80 | 134 | 5 | 973 | 191 | 0 | 101 | 1,514 | 959 | 264 | 9 | 0 |

Table S7. Training fold of the first iteration of the cross-validation.

| **Modality** | **Scanner** | | | | | | | |  | **Number of repetitions** | | | |
| --- | --- | --- | --- | --- | --- | --- | --- | --- | --- | --- | --- | --- | --- |
|  | **Aera** | **Avanto** | **Prisma** | **Sola** | **Signa**  **HDxt** | **Skyra** | **Titan**  **3T** | **Verio** | **TOTAL** | **1** | **2** | **3** | **4** |
| **MPRAGE** | 87 | 94 | 85 | 33 | 0 | 0 | 16 | 95 | 410 | 400 | 5 | 0 | 0 |
| **Synthetic MPRAGEs** | | | | | | | | | | | | | |
| **MPRAGE** | 87 | 93 | 86 | 32 | 0 | 0 | 19 | 97 | 414 | 404 | 5 | 0 | 0 |
| **2D T1w** | 154 | 277 | 132 | 19 | 0 | 0 | 60 | 232 | 874 | 339 | 260 | 5 | 0 |
| **2D T2w** | 230 | 302 | 148 | 33 | 0 | 0 | 68 | 234 | 1,015 | 291 | 342 | 12 | 1 |
| **2D T1wFLAIR** | 0 | 0 | 0 | 0 | 0 | 0 | 0 | 0 | 0 | 0 | 0 | 0 | 0 |
| **2D T2wFLAIR** | 141 | 52 | 123 | 33 | 0 | 0 | 48 | 48 | 445 | 429 | 8 | 0 | 0 |
| **2D T2wGRE** | 0 | 0 | 0 | 0 | 0 | 0 | 0 | 0 | 0 | 0 | 0 | 0 | 0 |
| **2D IR** | 0 | 0 | 0 | 0 | 0 | 0 | 0 | 0 | 0 | 0 | 0 | 0 | 0 |
| **TOTAL** | 699 | 818 | 574 | 150 | 0 | 0 | 211 | 706 | 3,158 | 1,863 | 620 | 17 | 1 |

Table S8. Linear correction subset of the first iteration of the cross-validation.

| **Modality** | **Scanner** | | | | | | | |  | **Number of repetitions** | | | |
| --- | --- | --- | --- | --- | --- | --- | --- | --- | --- | --- | --- | --- | --- |
|  | **Aera** | **Avanto** | **Prisma** | **Sola** | **Signa**  **HDxt** | **Skyra** | **Titan**  **3T** | **Verio** | **TOTAL** | **1** | **2** | **3** | **4** |
| **MPRAGE** | 19 | 44 | 20 | 6 | 0 | 0 | 7 | 35 | 131 | 129 | 1 | 0 | 0 |
| **Synthetic MPRAGEs** | | | | | | | | | | | | | |
| **MPRAGE** | 21 | 46 | 21 | 6 | 0 | 0 | 8 | 37 | 139 | 137 | 1 | 0 | 0 |
| **2D T1w** | 34 | 100 | 37 | 4 | 0 | 0 | 29 | 87 | 291 | 106 | 87 | 1 | 2 |
| **2D T2w** | 57 | 113 | 42 | 6 | 0 | 0 | 23 | 99 | 340 | 91 | 114 | 7 | 0 |
| **2D T1wFLAIR** | 0 | 0 | 0 | 0 | 0 | 0 | 0 | 0 | 0 | 0 | 0 | 0 | 0 |
| **2D T2wFLAIR** | 30 | 23 | 33 | 5 | 0 | 0 | 17 | 22 | 130 | 126 | 2 | 0 | 0 |
| **2D T2wGRE** | 0 | 0 | 0 | 0 | 0 | 0 | 0 | 0 | 0 | 0 | 0 | 0 | 0 |
| **2D IR** | 0 | 0 | 0 | 0 | 0 | 0 | 0 | 0 | 0 | 0 | 0 | 0 | 0 |
| **TOTAL** | 161 | 326 | 153 | 27 | 0 | 0 | 84 | 280 | 1,031 | 589 | 205 | 8 | 2 |

Table S9. Evaluation subset of the first iteration of the cross-validation.

| **Modality** | **Scanner** | | | | | | | |  | **Number of repetitions** | | | |
| --- | --- | --- | --- | --- | --- | --- | --- | --- | --- | --- | --- | --- | --- |
|  | **Aera** | **Avanto** | **Prisma** | **Sola** | **Signa**  **HDxt** | **Skyra** | **Titan**  **3T** | **Verio** | **TOTAL** | **1** | **2** | **3** | **4** |
| **MPRAGE** | 12 | 20 | 16 | 3 | 0 | 0 | 3 | 19 | 73 | 73 | 0 | 0 | 0 |
| **Synthetic MPRAGEs** | | | | | | | | | | | | | |
| **MPRAGE** | 13 | 17 | 16 | 3 | 0 | 0 | 2 | 18 | 69 | 69 | 0 | 0 | 0 |
| **2D T1w** | 27 | 48 | 28 | 2 | 0 | 0 | 11 | 52 | 168 | 58 | 52 | 2 | 0 |
| **2D T2w** | 37 | 54 | 28 | 3 | 0 | 0 | 16 | 49 | 187 | 58 | 61 | 1 | 1 |
| **2D T1wFLAIR** | 0 | 0 | 0 | 0 | 0 | 0 | 0 | 0 | 0 | 0 | 0 | 0 | 0 |
| **2D T2wFLAIR** | 22 | 13 | 24 | 3 | 0 | 0 | 11 | 11 | 84 | 82 | 1 | 0 | 0 |
| **2D T2wGRE** | 0 | 0 | 0 | 0 | 0 | 0 | 0 | 0 | 0 | 0 | 0 | 0 | 0 |
| **2D IR** | 0 | 0 | 0 | 0 | 0 | 0 | 0 | 0 | 0 | 0 | 0 | 0 | 0 |
| **TOTAL** | 111 | 152 | 112 | 14 | 0 | 0 | 43 | 149 | 581 | 340 | 114 | 3 | 1 |

Table S10. Training fold of the second iteration of the cross-validation.

| **Modality** | **Scanner** | | | | | | | |  | **Number of repetitions** | | | |
| --- | --- | --- | --- | --- | --- | --- | --- | --- | --- | --- | --- | --- | --- |
|  | **Aera** | **Avanto** | **Prisma** | **Sola** | **Signa**  **HDxt** | **Skyra** | **Titan**  **3T** | **Verio** | **TOTAL** | **1** | **2** | **3** | **4** |
| **MPRAGE** | 77 | 115 | 75 | 25 | 0 | 0 | 17 | 102 | 411 | 405 | 3 | 0 | 0 |
| **Synthetic MPRAGEs** | | | | | | | | | | | | | |
| **MPRAGE** | 79 | 113 | 76 | 25 | 0 | 0 | 20 | 104 | 417 | 409 | 4 | 0 | 0 |
| **2D T1w** | 141 | 282 | 137 | 15 | 0 | 0 | 72 | 247 | 894 | 337 | 264 | 7 | 2 |
| **2D T2w** | 210 | 314 | 140 | 25 | 0 | 0 | 79 | 259 | 1,027 | 285 | 346 | 14 | 2 |
| **2D T1wFLAIR** | 0 | 0 | 0 | 0 | 0 | 0 | 0 | 0 | 0 | 0 | 0 | 0 | 0 |
| **2D T2wFLAIR** | 122 | 64 | 117 | 24 | 0 | 0 | 55 | 52 | 434 | 422 | 6 | 0 | 0 |
| **2D T2wGRE** | 0 | 0 | 0 | 0 | 0 | 0 | 0 | 0 | 0 | 0 | 0 | 0 | 0 |
| **2D IR** | 0 | 0 | 0 | 0 | 0 | 0 | 0 | 0 | 0 | 0 | 0 | 0 | 0 |
| **TOTAL** | 629 | 888 | 545 | 114 | 0 | 0 | 243 | 764 | 3,183 | 1,858 | 623 | 21 | 4 |

Table S11. Linear correction subset of the second iteration of the cross-validation.

| **Modality** | **Scanner** | | | | | | | |  | **Number of repetitions** | | | |
| --- | --- | --- | --- | --- | --- | --- | --- | --- | --- | --- | --- | --- | --- |
|  | **Aera** | **Avanto** | **Prisma** | **Sola** | **Signa**  **HDxt** | **Skyra** | **Titan**  **3T** | **Verio** | **TOTAL** | **1** | **2** | **3** | **4** |
| **MPRAGE** | 30 | 22 | 31 | 9 | 0 | 0 | 5 | 29 | 126 | 126 | 0 | 0 | 0 |
| **Synthetic MPRAGEs** | | | | | | | | | | | | | |
| **MPRAGE** | 29 | 23 | 33 | 8 | 0 | 0 | 3 | 30 | 126 | 126 | 0 | 0 | 0 |
| **2D T1w** | 47 | 107 | 42 | 4 | 0 | 0 | 10 | 78 | 288 | 101 | 92 | 1 | 0 |
| **2D T2w** | 73 | 106 | 59 | 9 | 0 | 0 | 9 | 84 | 340 | 97 | 114 | 5 | 0 |
| **2D T1wFLAIR** | 0 | 0 | 0 | 0 | 0 | 0 | 0 | 0 | 0 | 0 | 0 | 0 | 0 |
| **2D T2wFLAIR** | 46 | 15 | 44 | 9 | 0 | 0 | 10 | 17 | 141 | 137 | 2 | 0 | 0 |
| **2D T2wGRE** | 0 | 0 | 0 | 0 | 0 | 0 | 0 | 0 | 0 | 0 | 0 | 0 | 0 |
| **2D IR** | 0 | 0 | 0 | 0 | 0 | 0 | 0 | 0 | 0 | 0 | 0 | 0 | 0 |
| **TOTAL** | 225 | 273 | 209 | 39 | 0 | 0 | 37 | 238 | 1,021 | 587 | 208 | 6 | 0 |

Table S12. Evaluation subset of the second iteration of the cross-validation.

| **Modality** | **Scanner** | | | | | | | |  | **Number of repetitions** | | | |
| --- | --- | --- | --- | --- | --- | --- | --- | --- | --- | --- | --- | --- | --- |
|  | **Aera** | **Avanto** | **Prisma** | **Sola** | **Signa**  **HDxt** | **Skyra** | **Titan**  **3T** | **Verio** | **TOTAL** | **1** | **2** | **3** | **4** |
| **MPRAGE** | 11 | 21 | 15 | 8 | 0 | 0 | 4 | 18 | 77 | 71 | 3 | 0 | 0 |
| **Synthetic MPRAGEs** | | | | | | | | | | | | | |
| **MPRAGE** | 13 | 20 | 14 | 8 | 0 | 0 | 6 | 18 | 79 | 75 | 2 | 0 | 0 |
| **2D T1w** | 27 | 36 | 18 | 6 | 0 | 0 | 18 | 46 | 151 | 65 | 43 | 0 | 0 |
| **2D T2w** | 41 | 49 | 19 | 8 | 0 | 0 | 19 | 39 | 175 | 58 | 57 | 1 | 0 |
| **2D T1wFLAIR** | 0 | 0 | 0 | 0 | 0 | 0 | 0 | 0 | 0 | 0 | 0 | 0 | 0 |
| **2D T2wFLAIR** | 25 | 9 | 19 | 8 | 0 | 0 | 11 | 12 | 84 | 78 | 3 | 0 | 0 |
| **2D T2wGRE** | 0 | 0 | 0 | 0 | 0 | 0 | 0 | 0 | 0 | 0 | 0 | 0 | 0 |
| **2D IR** | 0 | 0 | 0 | 0 | 0 | 0 | 0 | 0 | 0 | 0 | 0 | 0 | 0 |
| **TOTAL** | 117 | 135 | 85 | 38 | 0 | 0 | 58 | 133 | 566 | 347 | 108 | 1 | 0 |

Table S13. Training fold of the third iteration of the cross-validation.

| **Modality** | **Scanner** | | | | | | | |  | **Number of repetitions** | | | |
| --- | --- | --- | --- | --- | --- | --- | --- | --- | --- | --- | --- | --- | --- |
|  | **Aera** | **Avanto** | **Prisma** | **Sola** | **Signa**  **HDxt** | **Skyra** | **Titan**  **3T** | **Verio** | **TOTAL** | **1** | **2** | **3** | **4** |
| **MPRAGE** | 72 | 107 | 82 | 26 | 0 | 0 | 19 | 101 | 407 | 399 | 4 | 0 | 0 |
| **Synthetic MPRAGEs** | | | | | | | | | | | | | |
| **MPRAGE** | 76 | 106 | 84 | 25 | 0 | 0 | 19 | 103 | 413 | 407 | 3 | 0 | 0 |
| **2D T1w** | 135 | 291 | 125 | 16 | 0 | 0 | 68 | 263 | 898 | 330 | 274 | 4 | 2 |
| **2D T2w** | 208 | 322 | 148 | 26 | 0 | 0 | 67 | 271 | 1,042 | 304 | 346 | 14 | 1 |
| **2D T1wFLAIR** | 0 | 0 | 0 | 0 | 0 | 0 | 0 | 0 | 0 | 0 | 0 | 0 | 0 |
| **2D T2wFLAIR** | 123 | 60 | 120 | 25 | 0 | 0 | 49 | 62 | 439 | 423 | 8 | 0 | 0 |
| **2D T2wGRE** | 0 | 0 | 0 | 0 | 0 | 0 | 0 | 0 | 0 | 0 | 0 | 0 | 0 |
| **2D IR** | 0 | 0 | 0 | 0 | 0 | 0 | 0 | 0 | 0 | 0 | 0 | 0 | 0 |
| **TOTAL** | 614 | 886 | 559 | 118 | 0 | 0 | 222 | 800 | 3,199 | 1,863 | 635 | 18 | 3 |

Table S14. Linear correction subset of the third iteration of the cross-validation.

| **Modality** | **Scanner** | | | | | | | |  | **Number of repetitions** | | | |
| --- | --- | --- | --- | --- | --- | --- | --- | --- | --- | --- | --- | --- | --- |
|  | **Aera** | **Avanto** | **Prisma** | **Sola** | **Signa**  **HDxt** | **Skyra** | **Titan**  **3T** | **Verio** | **TOTAL** | **1** | **2** | **3** | **4** |
| **MPRAGE** | 30 | 33 | 27 | 9 | 0 | 0 | 5 | 33 | 137 | 135 | 1 | 0 | 0 |
| **Synthetic MPRAGEs** | | | | | | | | | | | | | |
| **MPRAGE** | 29 | 34 | 27 | 9 | 0 | 0 | 7 | 34 | 140 | 136 | 2 | 0 | 0 |
| **2D T1w** | 47 | 83 | 54 | 6 | 0 | 0 | 13 | 66 | 269 | 113 | 75 | 2 | 0 |
| **2D T2w** | 71 | 92 | 52 | 8 | 0 | 0 | 22 | 65 | 310 | 92 | 101 | 4 | 1 |
| **2D T1wFLAIR** | 0 | 0 | 0 | 0 | 0 | 0 | 0 | 0 | 0 | 0 | 0 | 0 | 0 |
| **2D T2wFLAIR** | 41 | 21 | 46 | 9 | 0 | 0 | 17 | 13 | 147 | 141 | 3 | 0 | 0 |
| **2D T2wGRE** | 0 | 0 | 0 | 0 | 0 | 0 | 0 | 0 | 0 | 0 | 0 | 0 | 0 |
| **2D IR** | 0 | 0 | 0 | 0 | 0 | 0 | 0 | 0 | 0 | 0 | 0 | 0 | 0 |
| **TOTAL** | 218 | 263 | 206 | 41 | 0 | 0 | 64 | 211 | 1,003 | 617 | 182 | 6 | 1 |

Table S15. Evaluation subset of the third iteration of the cross-validation.

| **Modality** | **Scanner** | | | | | | | |  | **Number of repetitions** | | | |
| --- | --- | --- | --- | --- | --- | --- | --- | --- | --- | --- | --- | --- | --- |
|  | **Aera** | **Avanto** | **Prisma** | **Sola** | **Signa**  **HDxt** | **Skyra** | **Titan**  **3T** | **Verio** | **TOTAL** | **1** | **2** | **3** | **4** |
| **MPRAGE** | 16 | 18 | 12 | 7 | 0 | 0 | 2 | 15 | 70 | 68 | 1 | 0 | 0 |
| **Synthetic MPRAGEs** | | | | | | | | | | | | | |
| **MPRAGE** | 16 | 16 | 12 | 7 | 0 | 0 | 3 | 15 | 69 | 67 | 1 | 0 | 0 |
| **2D T1w** | 33 | 51 | 18 | 3 | 0 | 0 | 19 | 42 | 166 | 60 | 50 | 2 | 0 |
| **2D T2w** | 45 | 55 | 18 | 8 | 0 | 0 | 18 | 46 | 190 | 44 | 70 | 2 | 0 |
| **2D T1wFLAIR** | 0 | 0 | 0 | 0 | 0 | 0 | 0 | 0 | 0 | 0 | 0 | 0 | 0 |
| **2D T2wFLAIR** | 29 | 7 | 14 | 7 | 0 | 0 | 10 | 6 | 73 | 73 | 0 | 0 | 0 |
| **2D T2wGRE** | 0 | 0 | 0 | 0 | 0 | 0 | 0 | 0 | 0 | 0 | 0 | 0 | 0 |
| **2D IR** | 0 | 0 | 0 | 0 | 0 | 0 | 0 | 0 | 0 | 0 | 0 | 0 | 0 |
| **TOTAL** | 139 | 147 | 74 | 32 | 0 | 0 | 52 | 124 | 568 | 312 | 122 | 4 | 0 |


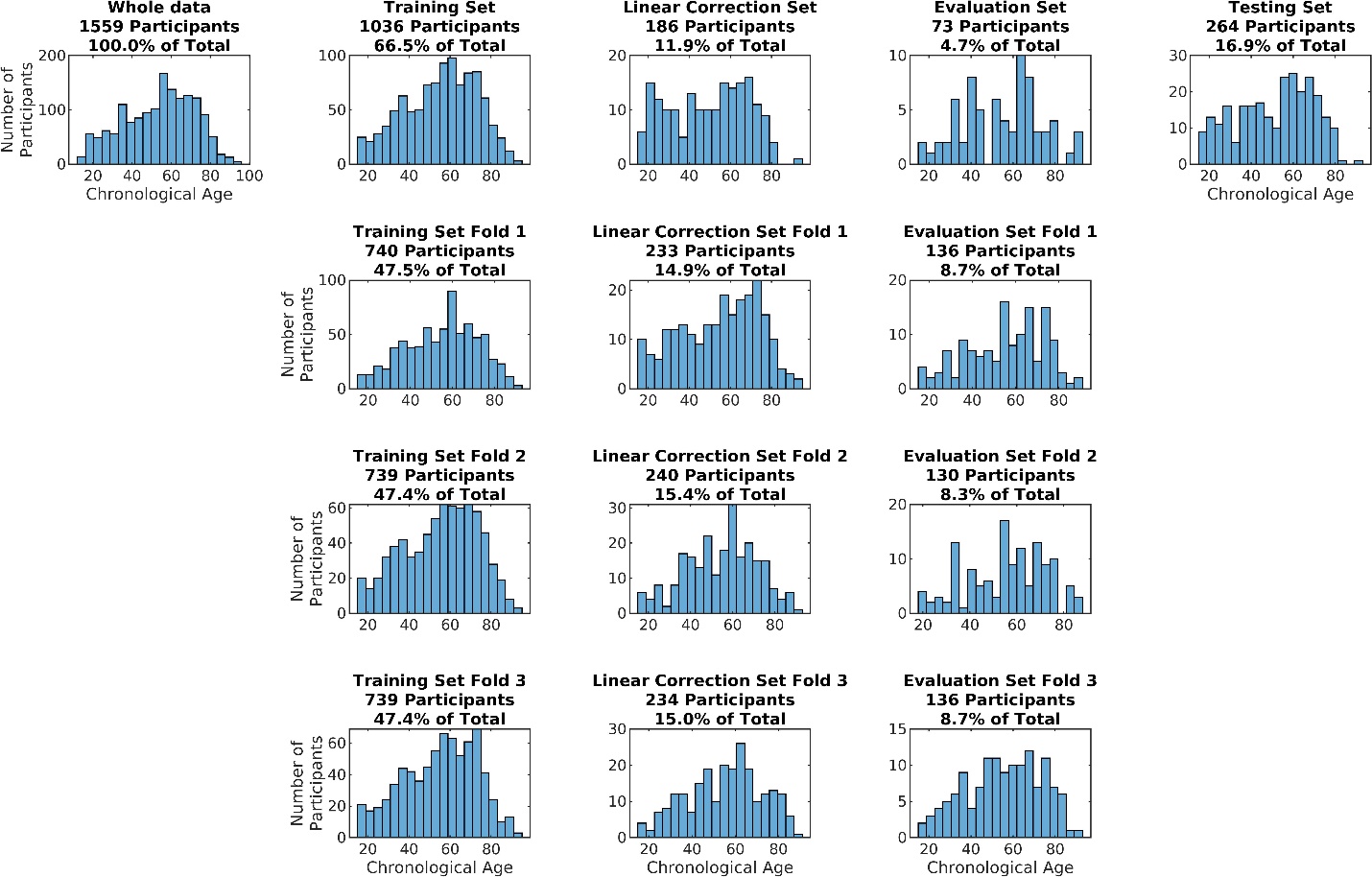


Figure S1. Distribution of chronological ages for the whole data and each split set of the data


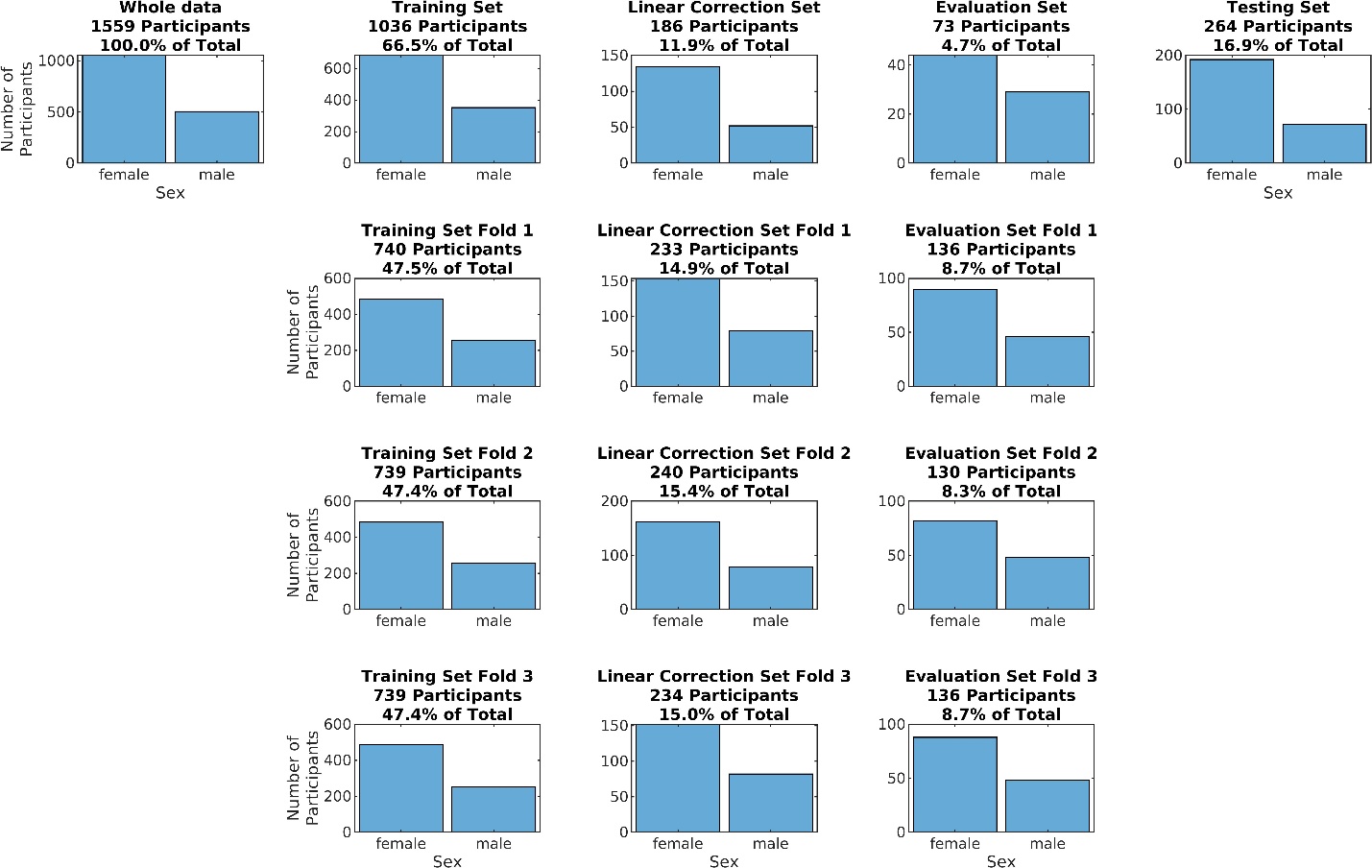


Figure S2. Distribution of sexes for the whole data and each split set of the data.


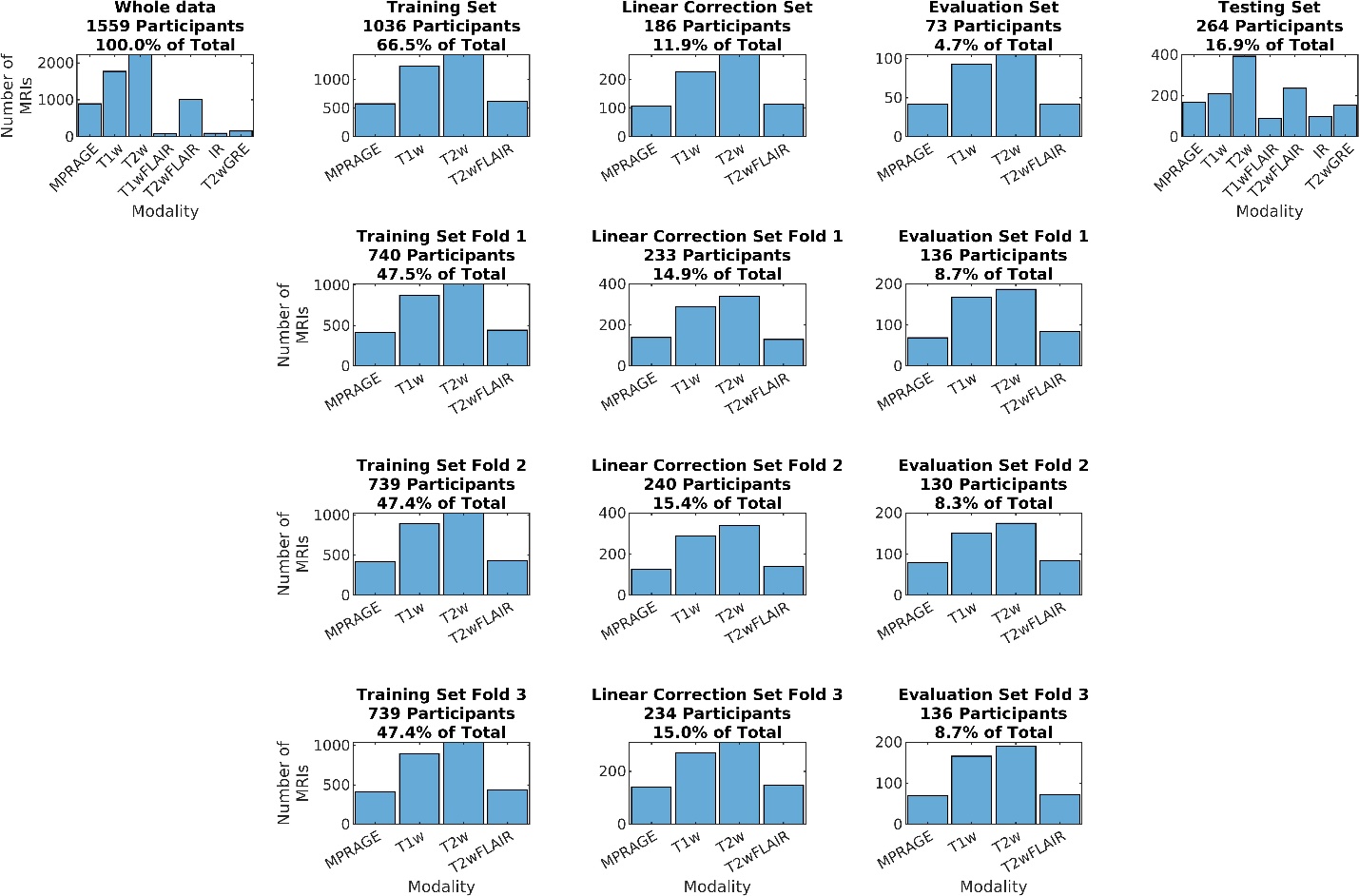


Figure S3. Distribution of modalities for the whole data and each split set of the data


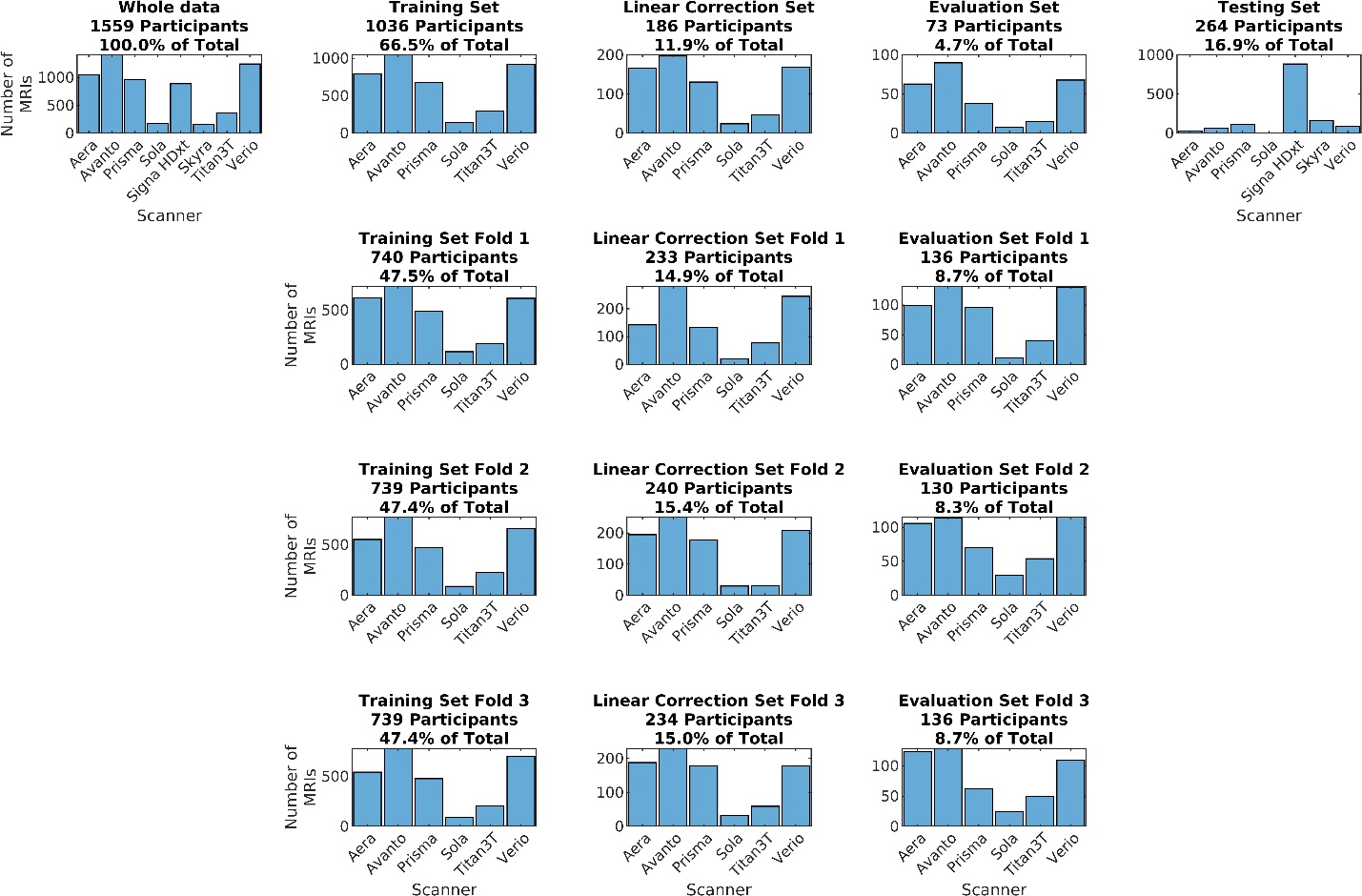


Figure S4. Distribution of scanner models for the whole and each split set of the data
